# ﻿Morphological and phylogenetic analysis of the early-diverging lineage of Glomeromycota suggest two new genera and recombinations in Archaeosporales

**DOI:** 10.3897/mycokeys.124.166449

**Published:** 2025-11-03

**Authors:** Keyvan Esmaeilzadeh-Salestani, Mariana Bessa de Queiroz, Vladimir Mikryukov, Sylwia Uszok, Bruno Tomio Goto, Leho Tedersoo, Franco Magurno

**Affiliations:** 1 Mycology and Microbiology Center, University of Tartu, Liivi 2, 50409 Tartu, Estonia; 2 Institute of Technology, University of Tartu, Nooruse 1, 50411 Tartu, Estonia; 3 Programa de Pós-graduação em Sistemática e Evolução, Centro de Biociências, Universidade Federal do Rio Grande do Norte, Campus Universitário, Natal 59072-970, RN, Brazil; 4 Institute of Ecology and Earth Sciences, University of Tartu, Liivi 2, 50409 Tartu, Estonia; 5 Institute of Biology, Biotechnology and Environmental Protection, Faculty of Natural Sciences, University of Silesia in Katowice, Jagiellońska 28, 40-032, Katowice, Poland; 6 Departamento de Botânica e Zoologia, Universidade Federal do Rio Grande do Norte, Campus Universitário, Natal 59072-970, RN, Brazil; 7 Department of Zoology, College of Science, King Saud University, 12371 Riyadh, Saudi Arabia

**Keywords:** Arbuscular mycorrhizal fungi, Archaeosporaceae, description of new taxa, molecular phylogeny, taxonomy

## Abstract

The family Archaeosporaceae (Archaeosporales), an early-diverging lineage of Glomeromycota, is currently represented by a single genus, *Archaeospora*, with seven species described. During the analysis of pot cultures established for the maintenance of Glomeromycota isolates, an unanticipated fungus emerged as a contaminant. Morphological and phylogenetic analyses revealed this fungus as a new species, forming an autonomous genus-level clade within Archaeosporaceae, herein proposed as *Antiquispora disseminans***gen. et sp. nov.** Sequences for this species were obtained using the newly designed primer FULlongF in combination with FULR, both not Glomeromycota specific. Positive clones after transformation were then screened and selected using the Archaeosporaceae-specific reverse primer SpAll_Archaeo_R in combination with the vector sequencing primers. In addition, independent phylogenetic analysis using specimen-based sequences and eDNA supported the genus status of *Archaeospora ecuadoriana* and *A. spainii*, both with diagnostic morphological traits, leading to the establishment of the new genus *Andinospora* to accommodate *Andinospora ecuadoriana***comb. nov.** and the genus status revalidation of *Palaeospora* with *P. spainii*. *Archaeospora* remains to include *A. trappei*, *A. europaea*, *A. schenckii*, while *A. myriocarpa* and *A. undulata* require additional analysis. Environmental sequences from the EUKARYOME database also showed that most of the genus-level clades described in Archaeosporaceae have worldwide distribution and are populated by several potential new species.

## ﻿Introduction

Arbuscular mycorrhizal fungi (AMF), mostly belonging to the phylum Glomeromycota, are ancient organisms that have coevolved with plants for over 400 million years, and associate with over 70% of terrestrial plant species (Brundrett and Tedersoo 2018). AMF plays a crucial role in several ecosystems by enhancing plant nutrient acquisition, improving water uptake, contributing to soil structure, and influencing plant community composition (Fall et al. 2022). Despite their ecological importance, their high diversity suggested by sequence-based environmental studies (Tedersoo et al. 2024a) and the recent description of several new species, only about 370 AMF species have been formally described, distributed across 52 genera and 21 families (da Silva et al. 2024, 2025; Goto et al. 2024; Błaszkowski et al. 2025a, b).

The family Archaeosporaceae (Archaeosporales) represents one of the earliest-diverging clades within Glomeromycota. It was established by Morton and Redecker (2001) with only one genus, *Archaeospora*, based on morphological and phylogenetic analyses of three species previously assigned to the genus *Acaulospora*: *Arc. trappei* (type species), which produces only acaulosporoid spores, and *Arc. leptoticha* and *Arc. gerdemannii*, both dimorphic - i.e., producing acaulosporoid and glomoid spores. Later, combining the morphology of the spore wall and the type of spore development (entrophosporoid), Sieverding and Oehl (2006) established the genus *Intraspora* within Archaeosporaceae to accommodate *I. schenckii*, previously classified as *Entrophospora schenckii* within the family Entrophosporaceae. Spain et al. (2006) also combined unique spore wall morphology and type spore development (acaulosporoid) to accommodate *Acaulospora appendicula*, *Appendicispora jimgerdemannii* and *Arc. gerdemannii* in a new genus *Appendicispora* in Archaeosporaceae. Walker et al. (2007a), using a molecular approach, described the genus *Ambispora*, which is the type genus for the family Ambisporaceae (formerly described as Appendicisporaceae) (Walker et al. 2007b) in Archaeosporales, accommodating *Amb. leptoticha*, *Amb. gerdemannii*, *Amb. fennica* and *Amb. callosa*, while only *Arc. trappei* remained in *Archaeospora* as previously suggested by Schüßler et al. (2001). Since *Appendicispora* represented a homonym of *Appendicospora* described previously by Hyde (1995) in Ascomycota, the names *Ambispora* and Ambisporaceae became legitimate (Walker 2008).

Following SSU rDNA sequence analysis, Schüßler and Walker (2010) transferred *I. schenckii* to *Archaeospora*, thereby synonymizing the genus *Intraspora*. Based on spore morphology and phylogeny of the ITS/LSU rDNA regions, Oehl et al. (2015) described the genus *Palaeospora*, with the type species *P. spainii*, forming a clade sister to *Arc. trappei* and *I. schenckii*. Subsequent analyses by Schüßler and Walker (2019) led to the reclassification of *P. spainii* into the genus *Archaeospora*, rendering *Palaeospora* a synonym of *Archaeospora*.

Morphologically, Archaeosporaceae is characterized by small, hyaline spores with distinctive developmental modes. While most species produce monomorphic acaulosporoid spores, some species are dimorphic by producing both acaulosporoid and glomoid spores, or, more rarely, also producing entrophosporoid spores (Sieverding and Oehl 2006). Oehl et al. (2011) transferred *Acaulospora myriocarpa* and *A. undulata* to *Archaeospora* considering their bi-walled spores, with both outer and inner walls composed of two-three layers each. *Archaeospora spainii* (formerly *Palaeospora*) is an exception in Archaeosporaceae, producing spores with three distinct two-layered walls (Oehl et al. 2015). The mycorrhizal structures consist of arbuscules and both intra- and extraradical hyphae, which generally stain weakly in Trypan blue; vesicles are rarely observed.

Currently, Archaeosporaceae is a monogeneric family represented by *Archaeospora* comprising seven species, of which *Arc. myriocarpa* and *Arc. undulata* (Oehl et al. 2011) lack phylogenetic characterization. However, the phylogenetic placement of *Arc. trappei*, the type species of the genus, remains uncertain, as sequences obtained from multiple isolates with similar morphology are found in separate clades (Schüßler and Walker 2019).

Recent advances in environmental DNA (eDNA) sequencing have revolutionized our understanding of microbial diversity, enabling the detection and identification of previously unknown fungal taxa directly from soil and root samples (Hug et al. 2016; Tedersoo et al. 2024b). Accordingly, Tedersoo et al. (2024a) highlighted two possible undescribed genera in Archaeosporaceae. While analyzing trap cultures intended for the maintenance of AMF isolates, we discovered an unexpected fungus that proliferated as a contaminant. Preliminary molecular analyses suggested that this fungus might be accommodated in one of the suggested new genera. Additionally, our investigations revealed the need for new combinations within the family. Therefore, the aims of this study are (i) to describe and characterize the morphology of the new fungus; (ii) to determine its position within Glomeromycota using both traditional and eDNA sequence-based approaches; (iii) to uncover candidate new species based on eDNA within the Archaeosporaceae; (iv) to revisit the genus *Palaeospora* with *P. spainii* based on morphological and molecular evidence; and (v) to accommodate *Arc. ecuadoriana* in a new genus within Archaeosporaceae.

## ﻿Materials and methods

### ﻿Origin of study material

*Antiquispora disseminans* pot culture was established in 2019 from spores collected in an old accession of *Diversispora epigaea* (formerly labeled as *Glomus versiforme*) available at the University of Turin (Italy). The substrate contained old spores (whose affiliation to *D. epigaea* was confirmed by rDNA sequencing) that had lost the ability to germinate, and smaller hyaline spores of unknown origin. About 100 spores were collected by F. Magurno and used to set up a pot culture with autoclaved sand and 5% bentonite as substrate, and *Plantago lanceolata* as host plant. After propagation, twelve single-spore pot cultures were also prepared under the same conditions. Pot cultures were maintained in the plant growth chamber at the Institute of Biology, Biotechnology and Environmental Protection (Katowice, Poland) and re-established approximately every six months upon verification of the presence of spores.

### ﻿Morphological analysis

Spores were isolated from pure culture pots by suspending 20–30 ml of substrate in water and passing the decanted supernatant through a 45-μm sieve. Spores were mounted on microscope slides using water, polyvinyl alcohol–lactic acid–glycerol (PVLG), and a mixture of PVLG and Melzer’s reagent (1:1, *v*/*v*). Morphological characteristics of spores were described from at least 100 spores, examined and photographed using dissecting and compound microscopes. The terminology of the spore structures is that presented in Błaszkowski (2012). Color descriptions were based on Kornerup and Wanscher (1983). The fungal nomenclature and the authors of fungal names were verified in the Index Fungorum database at www.indexfungorum.org (accessed on 24 February 2025). Voucher specimens were deposited in the UFRN–Fungos herbarium, Brazil (holotype) and in the Herbário Parque das Dunas RN, Brazil (isotype).

### ﻿Molecular analysis

Spores from both multi- and single-spore cultures were collected by wet sieving of the pot substrate. Genomic DNA was extracted with DNeasy PowerSoil Pro Kit (Qiagen, Hilden, Germany), according to the manufacturer’s instructions with modifications as in Magurno et al. (2024). Amplicons of SSU-ITS-LSU nrDNA partial genes were obtained by PCR with newly designed primer FULlongF (CCT AGT AAG CGT GAG TCA TCA) and FULR (Malicka et al. 2022) using the Phusion Plus DNA Polymerase (Thermo Fisher Scientific, Waltham, MA, USA) with a universal annealing temperature of 60 °C, according to the producer’s instructions. The annealing sites of the two primers are slightly internal to the positions of SSUmCfx and LSUmBrx (Krüger et al. 2009). Since these primers are not Glomeromycota-specific, clones positive for the insert were also checked with the Archaeosporaceae-specific reverse primer SpAll_Archaeo_R (CAT TAY GTC AGC ATC CTT G), in combination with the vector sequencing primers. Additional shorter sequences were obtained with the FULF/FULR primer pair as in Malicka et al. (2022). PCR amplicons were purified and cloned with GeneJET PCR Purification and CloneJET PCR Cloning Kits (Thermo Fisher Scientific, Waltham, MA, USA) and sequenced at Genomed S.A. (Warsaw, Poland). Sequences were deposited in GenBank with accession numbers PV873150–PV873156, PV938315–PV938337.

### ﻿Bioinformatic analysis

To infer the phylogenetic placement of the new species, a dataset was created including representatives of members of Archaeosporales, and Paraglomerales as the outgroup. More in detail, for Archaeosporaceae, the dataset comprised sequences from the new species and from all *Archaeospora* species (*sensu*Schüßler and Walker 2019) in possession of partial SSU-ITS-LSU nrDNA sequences or part of it, including sequences from twelve *Archaeospora trappei* isolates. The dataset was aligned with the online version of MAFFT v.7 (Katoh and Standley 2013) using the E-INS-i iterative refinement method (http://mafft.cbrc.jp/alignment/server/). Bayesian and maximum likelihood phylogenetic inference were performed via CIPRES Science Gateway 3.1 (Miller et al. 2010), using MrBayes v3.2.7 (Ronquist et al. 2012) and RAxML-NG (Kozlov et al. 2019) with partitions and nucleotide substitution models as described in Magurno et al. (2024). For Bayesian analysis, the number of generations was increased up to 5 million with a stop rule at split frequency standard deviation = 0.01. Phylogenetic trees from the two analyses were visualized, merged and edited in TreeGraph 2 (Stöver and Müller 2010). Clades were considered supported with Bayesian posterior probabilities ≥ 0.95 and ML bootstrap values ≥ 70%.

Due to the uncertainty of phylogenetic delimitation of *Arc. trappei*, we included a phylogenetic placement based on a comprehensive eDNA dataset (see description below). LSU_D2 sequences from 8 pure cultures of *Antiquispora disseminans* and sequences of *Arc. europaea*, *Arc. schenckii* and *Arc. ecuadoriana* were also added to the eDNA dataset alignment with the online version of MAFFT 7 using the “mafft --add”. The GTR+G+I model parameters of the reference tree were derived in RAxML using the function “--evaluate”. Sequences’ placement was achieved using EPA-ng (Barbera et al. 2019) and the resulting jplace file was converted to Newick format using Gappa.

A broader phylogeny, including a vast number of eDNA sequences, was conducted to confirm the phylogenetic placement of the new genus *Antiquispora* and detect candidate new species in Archaeosporaceae. Sequence data assigned to Glomeromycota were downloaded from three nucleotide sequence databases – EUKARYOME v.1.7 (Tedersoo et al. 2024b), NCBI (Sayers et al. 2024) and UNITE v.9.1 (Abarenkov et al. 2024). Unidentified fungi obtained from NCBI and UNITE were first assigned to rough taxonomic groups based on BLASTn queries against reference sequences in EUKARYOME v.1.7. Sequences affiliated with Archaeosporales were selected to assemble a SSU-ITS-LSU sequence dataset that was aligned using MAFFT v.7. The alignment was edited by manual trimming of overarching and misaligned ends and manual correction in case of obvious misalignments using AliView v.1.26 (Larsson 2014). In the alignment, at least one read from each described species was included to delimit clades and assign taxonomy.

The alignments were further filtered to exclude unalignable regions and processed in ClipKIT v.1.4.0 (Steenwyk et al. 2020) to remove phylogenetically uninformative positions. Finally, five partitions (SSU, ITS1, 5.8S, ITS2, LSU) were defined and employed in phylogenetic analyses. Maximum-likelihood tree reconstruction was performed using IQ-TREE v.2.2.5 (Minh et al. 2020), with a partitioned dataset under the GTR+I+G substitution model, including 1000 ultrafast bootstrap replicates and 1000 SH-aLRT tests. The trees were visualized and used for taxonomic re-annotation in FigTree v.1.4.4 (Rambaut 2018). The first three rounds of alignments and analyses were primarily used to detect and remove low-quality reads and chimeric sequences. From the fourth round onwards, high-quality reads were retained and used to generate final phylogenies for species and genus delimitation. To detect possible novel species clades, we used the following criteria: (i) monophyly; (ii) bootstrap support > 95; (iii) phylogenetic breadth and divergence roughly comparable to previously described taxa; and (iv) minimizing the number of novel taxa (i.e. preferably retaining larger groups if there were multiple alternative splitting possibilities). Intraspecies and interspecies divergences were calculated in Mothur 1.48 using the ‘calc=eachgap’ parameter. To produce the distance matrix, only species described in Archaeosporaceae with sequences overlapping the same SSU-ITS-LSU region were considered. The maximum intraspecific distance that did not exceed the minimum interspecies distance was selected as a threshold for OTU clustering.

Similarly, sequences of the eDNA dataset, covering the same target region, were used to produce a distance matrix, and then OTUs, according to the cutoff selected. For each OTU, sequences were mapped into the eDNA tree to detect possible species clades. Thereafter, the clades were evaluated based on the criteria described above.

Diagnoses of candidate novel species were prepared based on sequence motifs in the ITS and LSU regions by visually selecting the most distinguishing oligonucleotide barcodes of typically 20 bases using multiple sequence alignments. The barcodes were selected as not having ambiguous base calls (e.g., N or other IUPAC codes) for the suggested novel species and had at least two differences from closely related taxa. We also limited the number of permitted alignment mismatches (typically 0 or 1) for the candidate novel species to ensure species-level resolution.

Finally, metadata obtained from the EUKARYOME database were used to map the occurrences of members of Archaeosporaceae and their distribution across distinct biomes.

## ﻿Results

### ﻿Molecular data and phylogenetic analysis

Overall, seven partial SSU-ITS-LSU nrDNA and 23 partial LSU nrDNA clones were successfully sequenced from all the cultures obtained from multiple and single spores of *Antiquispora disseminans*. The highest dissimilarity between partial SSU-ITS-LSU nrDNA sequences was ca. 1%.

**Figure 1.**
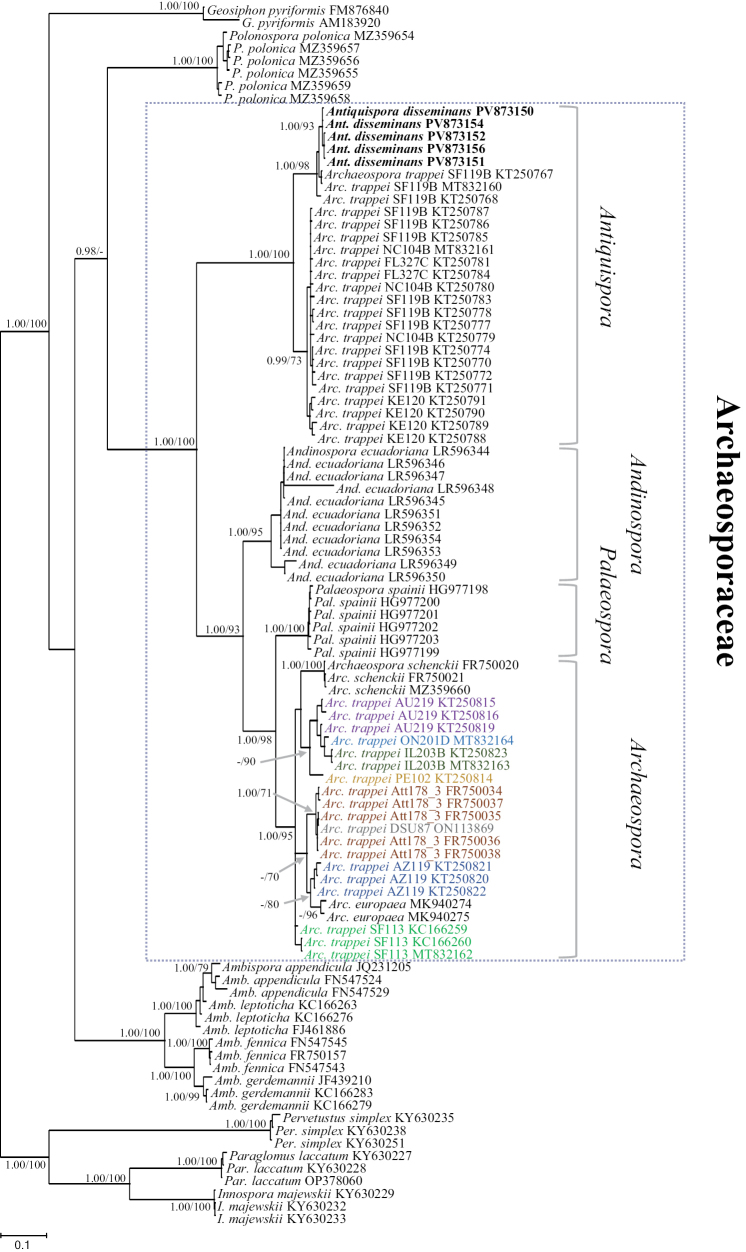
Phylogram generated from Maximum Likelihood (ML) and Bayesian Inference (BI) analyses displaying the phylogenetic relationships of *Antiquispora disseminans* among taxa in Archaeosporales. In the *Antiquispora* clade, sequences from isolates previously identified as *Arc. trappei*, are indicated with their original taxonomic affiliation. Colors in *Archaeospora trappei* (in *Archaeospora* clade) represent the different isolates, indicated before the accession numbers, from which sequences originated. Members of Paraglomerales were used to root the tree. Posterior probabilities and support values ≥ 0.98 and 70%, respectively, are indicated above or below the branches. Bar indicates 0.1 expected change per site per branch.

Phylogenies inferred using both Bayesian and Maximum Likelihood analysis recovered a similar topology (Fig. 1). The sequences of *Ant. disseminans* clustered in an autonomous, highly supported clade, shared with sequences from isolate SF119B (identified as *Archaeospora trappei*). Other sequences from this isolate and from the isolates FL327C, KE120, and NC104B (all from the INVAM collection) formed a sister clade with moderate support (0.99/73). Together, these two clades received full support (1.00/100) and were positioned as a sister group to the clade comprising all the other taxa in Archaeosporaceae. The analysis revealed fully or highly supported clades for *Arc. ecuadoriana*, *Arc. spainii*, and *Archaeospora* (sensu Oehl et al. 2015). The first two are here proposed as the basis for establishing the new genus *Andinospora* and for resurrecting the genus *Palaeospora*, respectively (Fig. 1). *Archaeospora trappei* sequences from eight isolates did not form a monophyletic clade but split into several clades with varying support, with each isolate’s sequences confined to a single clade.

The EPA analysis returned a slightly different topology, with most *Arc. trappei* sequences placed in two supported clades, or in their vicinity, corresponding to lineages called *Arc.* sp5 and *Arc. trappei* (named after the *Arc. trappei* isolate Att178-3, with SSU-ITS-LSU sequences available) in the eDNA-based phylogeny (Suppl. material 1). Sequences of *Arc. schenckii* and *And. ecuadoriana* were distributed across two distinct clades, whereas sequences of *Arc. europaea* clustered within the *Arc. trappei*. Finally, all 23 partial LSU rDNA sequences from 8 pure cultures of *Antiquispora disseminans* were placed in the corresponding species clade.

**Figure 2.**
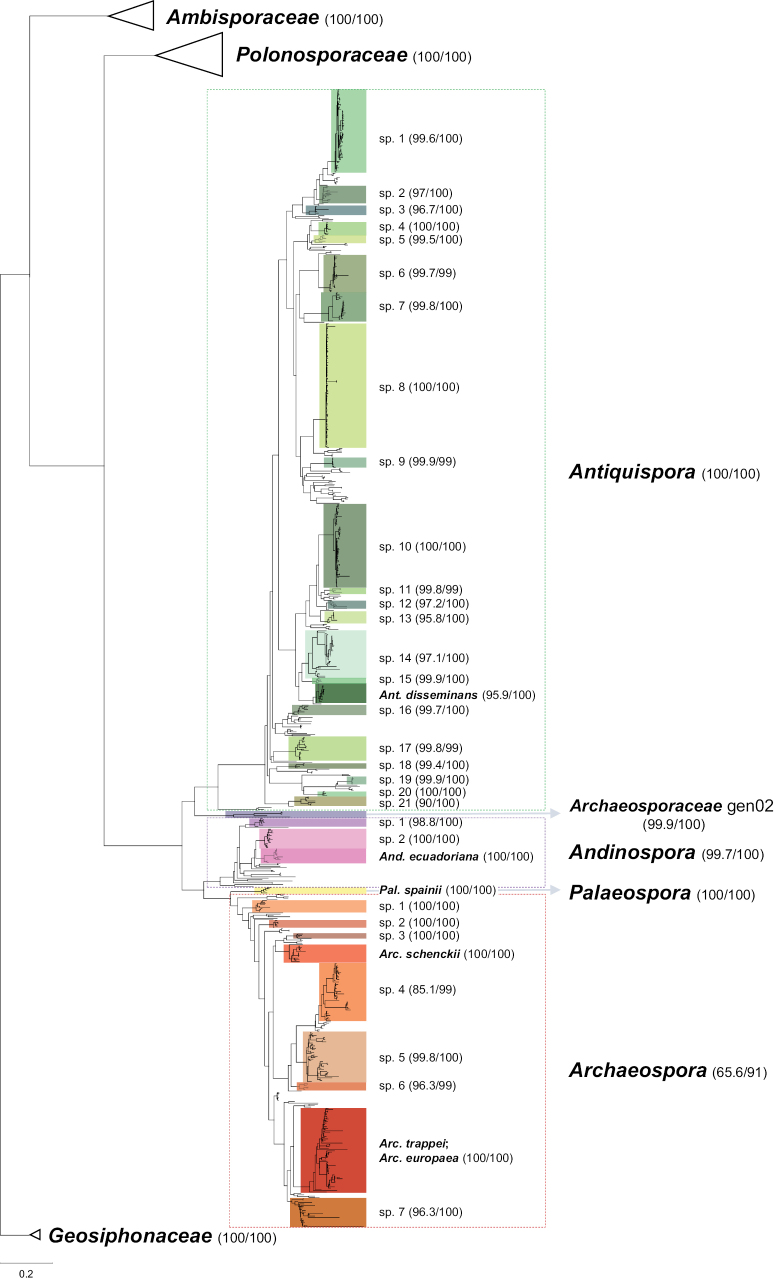
Phylogram generated from Maximum Likelihood analysis based on the eDNA dataset of Archaeosporales. The analysis conducted in IQ-TREE2 involved 918 sequences, 563 of which overlapped the SSU-ITS-LSU region, representative of four families and seven genera (plus one putative without representative isolates) in Archaeosporales. In Archaeosporaceae, candidate lineages at species rank are highlighted by colored boxes. Support values (SH-aLRT and Ultrafast Bootstrap support) are shown beside the respective lineage labels. Bar indicates 0.1 expected change per site per branch.

The phylogenetic analysis based on the eDNA database confirmed the support and autonomy of *Antiquispora*, *Andinospora*, *Palaeospora* and *Archaeospora* as genera in the Archaeosporaceae (Fig. 2, Suppl. material 2), with a topology coherent with the one in Fig. 1. The clade of *Archaeospora* was the only one to receive moderately strong support (91) as ultrafast bootstrap, and low support as SH-aLRT value (65.6). Additionally, a clade at genus rank (Archaeosporaceae_gen02), represented by a few sequences, was shown in a sister position to the clade containing the other genera.

**Figure 3.**
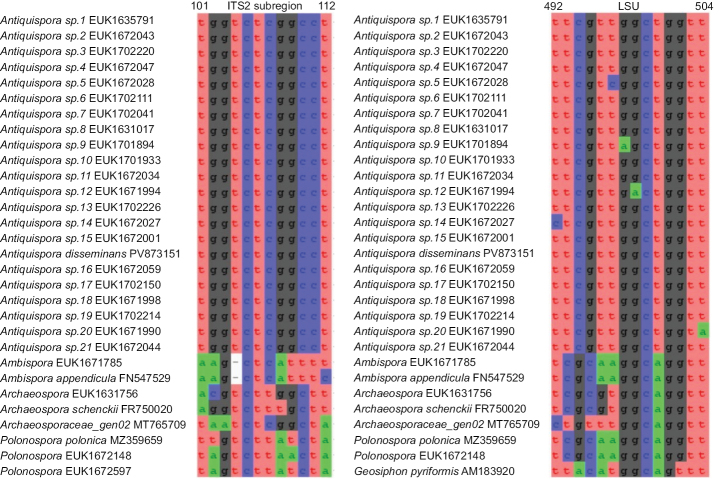
Separation of *Antiquispora* from other genera of Archaeosporaceae based on the ITS region (ITS2 positions 101–112 tggtctcggcct; one mismatch allowed) and LSU (positions 492–504 ttcgttggctggtt; one mismatch allowed).

Intra- and interspecies divergence analyses identified a 6% dissimilarity threshold, which was subsequently applied to delineate putative species-level lineages. *Antiquispora* comprised, besides *Ant. disseminans*, 21 well-supported lineages, which we suggest as candidate species pending further morphological and ecological characterization.

**Figure 4.**
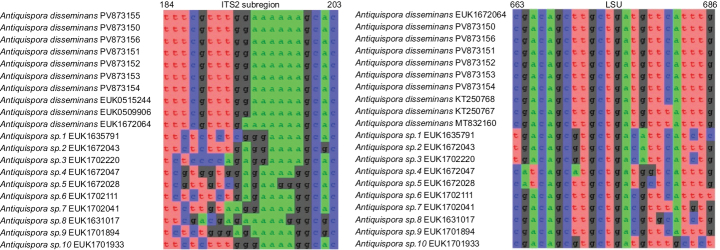
Separation of *Antiquispora disseminans* from other species of *Antiquispora* based on the ITS region (ITS2 positions 184–203 tttcgtttggaaaaaagcac; one mismatch allowed) and LSU (positions 663–686 cgacagcttgctgatgttcatttg; one mismatch allowed).

The motifs TGGTCTCGGCCTA and TTCGTTGGCTGGTT based on the ITS2 and LSU regions, respectively, as indicated in Fig. 3, were selected as diagnostic barcodes for the genus *Antiquispora*. Similarly, unique LSU barcodes were determined for all putative species in *Antiquispora* and ITS barcodes for 19 lineages (Fig. 4 for *Antiquispora disseminans*, Suppl. material 3 for the others).

Additionally, our analysis detected nine well-supported lineages in *Archaeospora*, including one represented by *Arc. schenckii* and one hosting the sequences of both *Arc. trappei* (isolate Att-178) and *Arc. europaea*. In *Andinospora*, two putative species lineages, distinct from *And. ecuadoriana*, were identified.

The phylogenetic distinctiveness of *Ant. disseminans*, *And. ecuadoriana*, and *P. spainii* as autonomous clades was also supported by blastn comparisons of their sequences with sequences of similar length to *Archaeospora*. The sequence divergences of *Ant. disseminans* from those of *Archaeospora*, *Andinospora*, and *Palaeospora*, were significantly greater (ca. 19–21%) than sequence divergences between other genera in Archaeosporaceae. The average sequence divergences between the genera *Antiquispora* vs. *Andinospora*, *Antiquispora* vs. *Archaeospora*, *Antiquispora* vs. *Palaeospora*, *Andinospora* vs. *Palaeospora*, *Andinospora* vs. *Archaeospora*, and *Palaeospora* v*s. Archaeospora* amounted to 19.5%, 21%, 21%, 13%, 14%, and 11.5%, respectively.

### ﻿Taxonomy

#### ﻿Description of new genera, new species and combinations

##### ﻿ Antiquispora

Taxon classificationFungiArchaeosporalesArchaeosporaceae

Magurno, Uszok, Esmaeilzadeh-Salestani, Tedersoo, M.B. Queiroz & B.T. Goto gen. nov.

5036281B-13F4-585F-8857-5A14626225DB

860980

Fig. 5A–I

###### Etymology.

Latin, *antiquus* (= ancient) and *spora* (= spores), referring to the phylogenetic placement of this genus within Archaeosporales, an early-diverging lineage of Glomeromycota.

###### Type species.

*Antiquispora disseminans* Magurno, Uszok, M.B. Queiroz & B.T. Goto.

###### Diagnosis.

Differs from *Archaeospora* and other genera of Archaeosporaceae in (i) having two hyaline spore walls and four layers, (ii) intraradical hyphae, spores, and vesicles stain darkly in trypan blue, and (iii) in the nucleotide composition of sequences of the partial SSU-ITS-LSU nrDNA region (see Discussion for details).

###### Genus description.

Spores acaulosporoid, formed singly in the substrate or occasionally within roots. Spores hyaline to white, small (55–67 µm diam), globose to subglobose, rarely ellipsoid to ovoid. Subcellular spore structure composed of two walls: the outer wall with two hyaline layers, and the inner wall with two permanent, flexible to semi-flexible layers. None of the layers in either wall stain with Melzer’s reagent. Sporiferous saccule hyaline to subhyaline, with a delicate mono- to bi-layered wall continuous with the two outer spore wall layers; usually collapsed or detached in extraradical spores. Spore walls and saccule wall staining dark in Trypan blue. Forming mycorrhizal structures staining dark in Trypan blue.

###### Ecology and distribution.

Environmental sequencing data indicate that the genus has a broad geographical and ecological distribution, having been recorded in tropical, subtropical, temperate, and even subpolar regions, across approximately 60 countries in the Americas, Africa, Asia, Europe, and Oceania (Suppl. material 4). It occurs in a broad range of natural and human-modified habitats, including various types of forests, shrublands, grasslands, woodlands, deserts, tundra, freshwater environments, and anthropogenic landscapes such as croplands, rangelands, villages, and urban areas (Suppl. material 5).

##### ﻿ Antiquispora disseminans

Taxon classificationFungiArchaeosporalesArchaeosporaceae

Magurno, Uszok, M.B. Queiroz & B.T. Goto sp. nov.

C4D2E308-0F90-5553-A5FA-9E8D27F28AA0

860982

Fig. 5A–I

###### Etymology.

Latin, *disseminans* (= dissemination), referring to the species capacity for rapid and successful propagation in culture pots.

###### Diagnosis.

Differs from *Archaeospora trappei* in (i) having two layers in the inner wall, whereas *A. trappei* has only one; by (ii) mycorrhizal structures (spores, hypha, and vesicles) staining dark in Trypan blue, and (iii) in the nucleotide composition of SSU-ITS-LSU nrDNA.

###### Species description.

**Figure 5.**
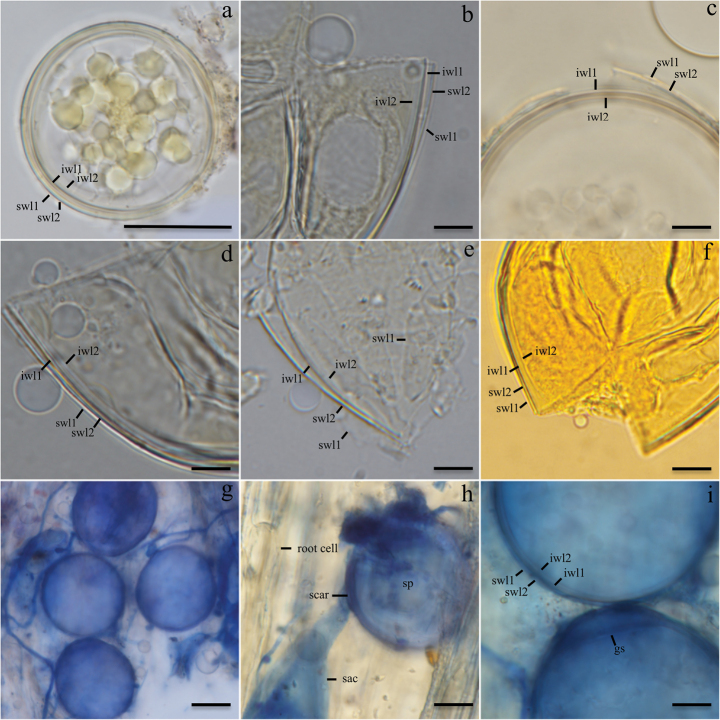
*Antiquispora disseminans*. A. Intact spores with dense, oily cytoplasmic content forming irregular droplets. B–F. Spores with two distinct walls (sw, iw), each composed of two layers: swl1–2 and iwl1–2; G. Spores and intraradical hyphae; H. Spore (sp) formed laterally on the neck of a sporiferous saccule (sac), with a detail of the scar at the spore base; I. Spores showing wall structure with four layers and detail of the germ shield (gs); C. Spores in PVLG. A, B, D–F. Spores in PVLG + Melzer’s reagent; G–I. Spores and other structures stained in 0.1% Trypan blue. Scale bars: 25 μm (A, G, H); 10 μm (B–F, I).

Acaulosporoid spores formed laterally on the neck of a sporiferous saccule (Fig. 5H), singly in the substrate or occasionally within roots. Spores hyaline to white (1A1), in maturity greyish white (1B1), globose to subglobose, (55–)60(–67) μm diam, rarely ellipsoid to ovoid (57–)62(–85) μm, with two walls (sw, iw) (Fig. 5A–F, I). Spore wall (sw) consists of two layers with 1.3–1.5 μm thick. Layer 1 (swl1) short-lived, evanescent, hyaline, thin, 0.3–0.5 μm thick. Layer 2 (swl2) permanent, laminated, hyaline to white (1A1), 0.8–1.0 μm thick. Inner wall (iw) consists of two, permanent, flexible to semi-flexible, (1.4–)1.5(–2.0) μm thick layers. Layer 1 (iwl1) uniform, slightly pigmented (1A2), 0.8–1.0 μm thick. Layer 2 (iwl2) hyaline, amorphous, 0.5–2.5 μm thick. None of the wall layers exhibits amyloid or dextrinoid reactions in Melzer’s reagent. Sporiferous saccule hyaline to subhyaline, subglobose to oblong, 15–55 × 30–75 µm diam, with an extremely delicate mono- to bi-layered wall continuous with the two outer layers of the outer spore wall (swl1-2) and coated with adherent granular material; rarely observable in spores outside roots due to frequent collapse or detachment during extraction from the soil, except in spores with adherent soil particles or remnant root fragments; occasionally visible within root tissues, where it stains dark with Trypan blue (Fig. 5H). The basal attachment point of the spore is marked by a semi-persistent cicatrix-like structure formed by the spore wall (Fig. 5H). The spore content consists of a dense, hyaline, oily substance forming unevenly distributed droplets that appear prominently darker than the spore walls, especially when observed in Melzer’s reagent (Fig. 5A). A germination shield (gs) forms from the inner wall (iw) (Fig. 5I). This structure is difficult to observe and was only visualized laterally in spores stained in Trypan blue. Mycorrhiza with hyphae, spores, and vesicles staining dark in Trypan blue (Fig. 5G–I). Sporocarps unknown. Glomoid spores not detected.

###### Specimen examined.

The material examined was obtained from culture pots containing the host *Plantago lanceolata* at the University of Silesia in Katowice, Poland, originally established for the maintenance of an inoculum of *Diversispora epigaea* sourced from Turin, Italy. However, the spores obtained represent a species distinct from that of the intended culture target, indicating unintentional contamination. The precise origin of this contaminant cannot be definitively established. Holotype: UFRN-Fungos 3785; Isotype: Herbário Parque das Dunas RN 10355.

###### Ecology and distribution.

In single-species cultures with *Plantago lanceolata*, *Ant. disseminans* formed typical arbuscular mycorrhizae, including spores, vesicles, and intra- and extraradical hyphae. This species exhibited an extraordinarily high success rate in pot cultures, with approximately two-thirds of single-spore cultures resulting in abundant spore production. Based on environmental DNA sequences, *Ant. disseminans* occurs in a variety of habitats, indicating a broad distribution. It has been detected in subtropical coniferous forest in Saltillo, Mexico; in urban soil environments in the United States; and in temperate woodland soil in Viru-Jaagupi, Estonia. These findings suggest that the species is ecologically versatile and capable of thriving across diverse climatic and habitat conditions.

##### ﻿ Andinospora

Taxon classificationFungiArchaeosporalesArchaeosporaceae

Magurno, Uszok, Esmaeilzadeh-Salestani, Tedersoo, M.B. Queiroz & B.T. Goto gen. nov.

E163984B-AD6E-5C0B-8B32-E1F59E5205E7

MB 860983

###### Etymology.

Latin, *Andinus* (= referring to the Andes Mountain Range), where the species was originally found, and *spora* (= spores).

###### Type genus.

*Andinospora ecuadoriana* (A. Schüßler & C. Walker) Magurno, Uszok, M.B. Queiroz & B.T. Goto, comb. nov.

###### Basionym.

*Archaeospora ecuadoriana* A. Schüßler & C. Walker, Mycorrhiza 29: 437 (2019).

###### Diagnosis.

Differs from *Archaeospora* and other genera of Archaeosporaceae in (i) having a spore wall with one layer and an inner wall two-layered, and (ii) in the nucleotide composition of sequences of the SSU-ITS-LSU nrDNA region (see Discussion for details).

###### Genus description.

Spores formed singly in soil, roots, or small clusters, laterally, or intercalary to the sporiferous saccule. Hyaline, small (20 µm diam), glomoid spores detected. Acaulosporoid and entrophosporoid spores hyaline, globose, subglobose, broadly ellipsoid, ovoid, obovoid, or irregular, 43–77 × 43–99 µm diam. Spore wall one-layered continuous with sporiferous saccule wall layer, and an inner (germinal) wall with two hyaline permanent layers. Sporiferous saccule hyaline to subhyaline, with a mono-layered wall continuous with the laminated spore wall layer. Spore walls staining in Trypan blue. Forming mycorrhizal structures staining in Trypan blue.

###### Ecology and distribution.

Environmental sequencing data indicate that the genus has been recorded in about 15 countries across Africa, South America, Oceania, Europe, and Asia (Suppl. material 6). Most records come from tropical and subtropical ecosystems, particularly broadleaf and coniferous forests, but have also been found in temperate grasslands and woodlands (Suppl. material 5).

##### ﻿ Andinospora ecuadoriana

Taxon classificationFungiArchaeosporalesArchaeosporaceae

(A. Schüßler & C. Walker) Magurno, Uszok, Esmaeilzadeh-Salestani, Tedersoo, M.B. Queiroz & B.T. Goto comb. nov.

92ADDE7E-4DAC-5C83-9A46-861E9D17D637

MB 860984

###### Species description.

As in *Andinospora* description.

###### Ecology and distribution.

The species showed a broad ecological distribution, having been detected across multiple continents, including Australia, South America, Africa, Asia, and Central America. It occurs in a wide range of biomes, such as temperate and subtropical broadleaf forests, tropical forests, tropical coniferous forests, and both temperate and tropical woodlands. This widespread occurrence across diverse climatic zones and vegetation types suggests the species is ecologically versatile and capable of thriving under varied environmental conditions, possibly reflecting a broad host range or high functional adaptability.

##### ﻿ Archaeospora

Taxon classificationFungiArchaeosporalesArchaeosporaceae

(J.B. Morton & D. Redecker) emend. Magurno, Uszok, Esmaeilzadeh-Salestani, Tedersoo, M.B. Queiroz & B.T. Goto

DE2FAA34-EE2A-54D7-A285-3D52980C6DE7

###### Genus description.

Spores acaulosporoid, entrophosporoid and/or glomoid formed singly or in aggregates in the substrate or occasionally within roots. Acaulosporoid and entrophosporoid spores hyaline or white to light yellow, small (22–114 µm diam), globose to subglobose, rarely ellipsoid to ovoid. Subcellular spore structure composed of two walls: the outer wall with one-two hyaline layers, and the inner wall with one to three permanent, flexible to semi-flexible layers. None of the layers in either wall stain with Melzer’s reagent. Sporiferous saccule hyaline to subhyaline, with a delicate mono- to bi-layered wall continuous with the two outer spore wall layers; usually collapsed or detached in extraradical spores. Glomoid spores hyaline to white, small (22–31 µm diam), with a bi-layered wall. Forming mycorrhizal structures with weak reaction in Trypan blue.

###### Type genus.

*Archaeospora trappei* (R.N. Ames & Linderman) J.B. Morton & D. Redecker, Mycologia 93 (1): 183 (2001).

###### Basionym.

*Acaulospora trappei* R.N. Ames & Linderman, Mycotaxon 3 (3): 566 (1976).

###### Other species.

*Archaeospora europaea* Oehl, Palenz., Sánchez-Castro, V.M. Santos & G.A. Silva, Sydowia 71: 131 (2019).

*Archaeospora myriocarpa* (Spain, Sieverd. & N.C. Schenck) Oehl, G.A. Silva, B.T. Goto & Sieverd., Mycotaxon 117: 430 (2011).

*Archaeospora schenckii* (Sieverd. & S. Toro) C. Walker & A. Schüßler, The Glomeromycota: a species list with new families and new genera: 53 (2010).

*Archaeospora undulata* (Sieverd.) Sieverd., G.A. Silva, B.T. Goto & Oehl, Mycotaxon 117: 430 (2012).

###### Ecology and distribution.

Environmental sequencing data show that the genus has a broad distribution, with records from 38 countries across tropical, subtropical, temperate, and subpolar regions in Africa, the Americas, Europe, Asia, and Oceania (Suppl. material 7). It has been detected in diverse biomes, including broadleaf and coniferous forests, woodlands, grasslands (montane and flooded), shrublands, deserts, freshwater river systems, and a variety of anthropogenic habitats such as croplands, rangelands, villages, and urban areas (Suppl. material 5).

## ﻿Discussion

Environmental DNA (eDNA) metabarcoding is increasingly recognized as a powerful tool for biodiversity assessment, allowing the discovery of putative novel taxa across diverse ecosystems (Tedersoo et al. 2024a). Its application has expanded from general biodiversity monitoring to the detection of non-indigenous species (Duarte et al. 2021), agricultural pests (Kestel et al. 2022), and rewilding outcomes (Cowgill et al. 2025). A key advantage of eDNA lies in its sensitivity and cost-effectiveness compared to traditional methods (Fediajevaite et al. 2021), enabling the detection of rare or cryptic species. Nevertheless, caution is advised, as intragenomic variation in rDNA markers can significantly undermine species delimitation and identification when paralogous sequences with conflicting signals are included in the analysis (Magurno et al. 2024; Corradi et al. 2025). While eDNA holds great promise for uncovering undescribed biodiversity, its full potential will depend on continued methodological refinement and validation against established taxonomic frameworks.

Tedersoo et al. (2024a) presented a comprehensive Glomeromycota phylogeny based on eDNA, identifying numerous potentially new taxa at various ranks. Following this lead, in the present paper we put a face, or rather, a spore, on one of those genera (namely Archaeosporaceae_gen01) suggested in Archaeosporaceae.

Our morphological and phylogenetic analysis supported *Antiquispora disseminans* as a new species in an autonomous fully supported genus-level clade in Archaeosporaceae. The position of members of this species was already shown in Schüßler and Walker (2019) when *Archaeospora ecuadoriana* was described and *Palaeospora* was synonymised with *Archaeospora*. However, no formal action was taken since the INVAM isolates (SF119B, FL327C, KE120, NC104B) were morphologically identified as *Arc. trappei*. One isolate (NC104B: NORTH CAROLINA: Dill’s Creek wetland, 1993) was also among the specimens analyzed for the synonymization of *Acaulospora trappei* in *Arc. trappei* (Morton and Redecker 2001). Importantly, the position of sequences from these isolates, whose clade branched before the *And. ecuadoriana* clade, was used to further support the synonymization of *Palaeospora* due to its nested position within *Archaeospora*.

In light of these findings, the taxonomic status of *Arc. ecuadoriana* and *Arc. spainii* have been revisited, according to their phylogenetic position and morphology, as a new genus *Andinospora* and the reestablishment of *Palaeospora*, respectively. The morphological features distinguishing *Ant. disseminans* are limited (enough to mislead the previous identification of the species), due to the similar morphology of spore wall in species belonging to those orders referred to as basal in the Glomeromycota. Nevertheless, the percentage of identity between its sequences and those of members of *Archaeospora*, *Andinospora* and *Palaeospora* unequivocally justified its taxonomic status as separate genera.

Interestingly, none of the 23 sequences obtained from eight pure cultures were placed in the *Antiquispora* sp14 clade, populated by sequences of isolates SF119B, FL327C, KE120, NC104B. Considering also the high dissimilarity between sequences of *Ant. disseminans* and those from this clade (ca. 11%), we opted for considering *Antiquispora* sp14 as a distinct putative species. How sequences reportedly derived from SF119B populated both clades remains unexplained, with possibilities of misbehaving paralogs and mislabeled cultures.

Previous phylogenetic analysis showed that sequences from different isolates of *Arc. trappei* are scattered along the *Archaeospora* clade (Schüßler and Walker 2019; Błaszkowski et al. 2021). This confusing pattern was confirmed in our analysis when phylogeny was conducted employing only specimen-derived sequences (Fig. 1). A more coherent phylogeny was obtained using hundreds of eDNA sequences that resulted in phylogenetic placement of most *Arc. trappei* sequences within two large clades (Suppl. material 1). Considering the outcome of both analyses and the extrapolated criterion used to suggest lineages, several (not mutually exclusive) interpretations can be suggested: (i) *Arc. trappei* sequences belong to two species and *Arc. europaea* has to be considered as a synonym of one of them; (ii) *Archaeospora* hosts several cryptic species resembling the features of *Arc. trappei*, and *Arc. europaea* remains a separate, valid species; (iii) the rDNA marker alone is not able to resolve the phylogeny of *Archaeospora* or *Arc. trappei* complex; (iv) the phylogeny of *Archaeospora* is strongly disturbed by paralog sequences, causing polyphyletic signals as in other families of Glomeromycota (Błaszkowski et al. 2022; Magurno et al. 2024; Corradi et al. 2025). The placement of *Arc. europaea* within an *Arc. trappei* clade should be viewed with caution. In addition to morphological discrepancies, the lack of LSU coverage in both *Arc. europaea* and its closest eDNA matches may have introduced biases.

The taxonomic confusion surrounding Archaeosporaceae species stems from a long history of complex classifications due to similarities of spore development and spore wall structure. All seven previously described species share several features, including small, hyaline, acaulosporoid/entrophosporoid spores with 3–4 layers in the walls (Ames and Linderman 1976; Spain 2003; Hafeel 2004; Błaszkowski and Czerniawska 2005; Sieverding and Oehl 2006). The only exception is *Palaeospora spainii*, with eight layers distributed in three distinct walls. *Andinospora* and *Antiquispora* share a similar spore development with *Archaeospora*, characterized by (i) small (<100 μm), hyaline, spores produced lateral or intercalary to sporiferous saccule; (ii) spore wall with two walls, an outer (sw) and inner (iw) wall producing a delicate germinal shield. However, *Palaeospora* present a distinctive spore development, characterized by (i) small (<100 μm), hyaline, spores produced laterally to sporiferous saccule, and (ii) spore wall with three walls, an outer, middle, and inner (germinal) wall with eight layers, resembling *Acaulospora* species but without a beaded layer (Sieverding and Oehl 2006). Also considering spore wall organization and phylogenetic analysis Błaszkowski et al. (2021) describe a new family, Polonosporaceae in Archaeosporales with a new genus *Polonospora* based on species previously described as *Acaulospora polonica*.

A comparative overview of spore type, number of walls and wall layers, and the mycorrhizal structures, including their staining patterns in Trypan blue, observed in all species of Archaeosporaceae is summarized in Table 1. Traits such as spore type and the number of inner wall layers proved inconclusive for distinguishing species, particularly within *Archaeospora*. Moreover, numerous inconsistencies in the number of inner wall layers are common in the *Archaeospora* literature, often arising from descriptions based on different isolates. These discrepancies may indicate either (i) that these isolates correspond to different species, or (ii) that a single species exhibits high morphological variation. Based on the current evidence, we consider the first explanation to be more plausible. Therefore, for morphological comparisons, we prioritize the original descriptions or redescriptions that include examination of type specimens.

**Table 1.** Comparison of spore type, number of walls and wall layers and mycorrhizal structures among species of the family Archaeosporaceae.

Species	Spore type	Number of spore walls	Number of layers (sw)	Number of layers (iw)	Mycorrhizal structures/ Trypan blue staining
*Palaeospora spainii*	Acaulosporoid/ Glomoid	3 (sw, mw, iw)	3	5	Arbuscules, intra- and extraradical hyphae do not stain or stain weakly. Vesicles absent.
*Andinospora ecuadoriana*	Acaulosporoid/ Entrophosporoid/ Glomoid	2 (sw, iw)	1	2	Spores and intraradical hyphae staining weakly to moderately.
*Antiquispora disseminans*	Acaulosporoid	2 (sw, iw)	2	2	Spores, intra- and extraradical hyphae, and vesicles stain darkly.
*Archaeospora trappei*	Acaulosporoid	2 (sw, iw)	2	1	Arbuscules, intra- and extraradical hyphae stain weakly.
*Archaeospora schenckii*	Entrophosporoid	2 (sw, iw)	2	1 or 3	Arbuscules vesicles and intraradical hyphae stain weakly, whereas the inner spore wall stains darkly.
*Archaeospora europaea*	Acaulosporoid/ Glomoid	2 (sw, iw)	2	3	Arbuscules, intra- and extraradical hyphae not or stain weakly. Vesicles absent.
*Archaeospora myriocarpa*	Acaulosporoid	2 (sw, iw)	2	1	Intraradical hyphae stain weakly. Typical arbuscules and vesicles absent.
*Archaeospora undulata*	Acaulosporoid	2 (sw, iw)	2	1	Unknown

*Abbreviation of spore walls and spore wall layers: spore wall (sw), middle wall (mw) and inner wall (iw) with 1–3 layers.

The re-establishment of *Palaeospora* with *P. spainii* is supported by morphological features of the spore wall, as originally suggested by Oehl et al. (2015). *Palaeospora spainii* remains the only species in Archaeosporaceae known to possess eight layers in three spore walls (a feature observed in other Archaeosporales families such as Ambisporaceae and Polonosporaceae), while other members of *Archaeospora* typically exhibit two spore walls (Spain et al. 2006; Błaszkowski et al. 2021). The new genera *Antiquispora* and *Andinospora* within Archaeosporaceae also exhibit spores with two walls. Although *And. ecuadoriana* was described by Schüßler and Walker (2019) as having “three wall components (C1-C3)”, this terminology deviates from the standard wall-and-layer nomenclature (Błaszkowski 2012). Our reassessment of the published micrographs indicates that spores of *And. ecuadoriana* presents the typical bi-walled structure (see fig. 2h, k, m, n in Schüßler and Walker 2019), where C1 represents a single-layered outer wall and C2-C3 correspond to the bi-layered inner (germinal) wall (see fig. 2i, n in Schüßler and Walker 2019).

*Andinospora ecuadoriana* differs from *Archaeospora* species by having a single-layered spore wall, whereas species of *Archaeospora* present a two-layered spore wall. Additionally, *And. ecuadoriana* has a two-layered inner wall, whereas *Archaeospora* species typically have a single-layered inner wall. *Archaeospora europaea* is the only *Archaeospora* species described with five wall layers, including three of these in the inner wall. Although *Arc. schenckii* was initially described as having three wall layers, Sieverding and Oehl (2006), upon reexamining the type material, concluded that it also possesses five layers.

*Antiquispora disseminans* differs from *Andinospora* by having a two-layered outer wall (vs. a single-layered), and from *Archaeospora* species by having a two-layered inner wall (vs. one- or three-layered). However, the most distinctive feature of *Antiquispora* is the staining of its mycorrhizal structures in Trypan blue. While *Archaeospora* and *Palaeospora* generally do not stain or stain only faintly, and *Andinospora* exhibits slight staining, *Antiquispora* shows consistently dark staining. In addition, the sporiferous saccule of *Antiquispora* stains similarly to the spores, whereas in *Andinospora* the saccule stains weakly and in *Arc. schenckii* shows no staining. For other species in the family, information on saccule staining is currently unavailable.

Based on our analysis and results, we follow the recommendations to bi-morphic species proposed by Błaszkowski et al. (2022) for the upcoming works involving species in the Archaeosporaceae that (i) *Arc. trappei*-like culture collections should be carefully described to characterize the spore wall structure, germinal shield (or orb) and the possible presence of dimorphism, (ii) the morphology of *Arc. trappei*-like and/or glomoid-like spores should be described to present possible differences, (iii) descriptions of new species based on *Arc. trappei*-like cultures should be based on morphological specimens-based approaches with a strong phylogenetic dataset to clarify potential new species in the clade, (iv) spore wall description should apply standardized terminology to spore wall organization, avoiding confusion in spore wall interpretations, and finally (v) staining spores and mycorrhizal structures with Trypan blue must be included in species descriptions and characterization of isolates, since it often provides more informative results than Melzer’s reagent, which frequently gives weak or irrelevant reactions within this group.

Finally, the large number of sequences retrieved from the Eukaryome database, along with the associated metadata, provided a powerful tool to map the occurrences of Archaeosporaceae members and to shed light on their ecology and distribution. *Antiquispora* and *Archaeospora* accounted for 250 and 221 occurrences (sample IDs in the database), respectively, while *Rhizoglomus*, one of the most widespread genera in Glomeromycota, was found in 889. *Funneliformis*, to provide another example, was recorded in “only” 203 samplings.

When scaling up one taxonomic rank, the family Archaeosporaceae accounted for 429 occurrences, compared to 2727 for Glomeraceae*sensu*Schüßler and Walker (2010). Based on these data, *Antiquispora* appears to be not only quite common but also the most widespread genus within Archaeosporaceae.

The reason it has been “hidden” until now remains unclear, but mismatches in Glomeromycota-specific primers commonly employed in environmental studies might be one of the culprits. Indeed, most sequences originated from studies using universal fungal primers, which could have helped overcome such primer bias. Similarly, non-Glomeromycota-specific primers were used to obtain the sequences from the *Antiquispora disseminans* isolate.

## Supplementary Material

XML Treatment for Antiquispora

XML Treatment for Antiquispora disseminans

XML Treatment for Andinospora

XML Treatment for Andinospora ecuadoriana

XML Treatment for Archaeospora

## ﻿Additional information

### Use of AI

No use of AI was reported.

### Author ORCIDs

Keyvan Esmaeilzadeh-Salestani https://orcid.org/0000-0002-6882-7616

Mariana Bessa de Queiroz https://orcid.org/0000-0003-0197-7203

Vladimir Mikryukov https://orcid.org/0000-0003-2786-2690

Sylwia Uszok https://orcid.org/0000-0001-5961-7695

Bruno Tomio Goto https://orcid.org/0000-0001-6157-4954

Leho Tedersoo https://orcid.org/0000-0002-1635-1249

Franco Magurno https://orcid.org/0000-0002-3117-8149

### Data availability

All of the data that support the findings of this study are available in the main text or Supplementary Information.

## Supplementary materials

Supplementary material 1 Snapshot of EPA analysis to infer the position of *Archaeospora trappei*, *Arc. schenckii*, *Arc. europaea*, *Andinospora ecuadoriana* and *Antiquispora disseminans* isolates in the reference eDNA tree Data type pdf Explanation note Names of isolates are labeled in color. Accession numbers: *Archaeospora trappei* Att178_3 (FR750034, FR750035, FR750036, FR750038, FR750037), AU219 (KT250815, KT250816, KT250819), SF113 (KC166259, KC166260, MT832162), AZ119 (KT250821, KT250820, KT250822), ON201D (MT832164), PE102 (KT250814), IL203B (KT250823, MT832163), DSU87 (ON113869). *Archaeospora schenckii* Att212-4 (FR750020, FR750021), CL401 (KT250807, KT250792, KT250802, KT250812, KT250797, KT250811, KC166258, KT250813). *Archaeospora europaea* SAF1151 (MK940274, MK940275). *Andinospora ecuadoriana* N2 (LR596344, LR596345, LR596349, LR596350, LR596351, LR596352, LR596353, LR596354), N6 (LR596346, LR596347, LR596348). *Antiquispora disseminans* 1 (PV938315, PV938316, PV938317), 3 (PV938327, PV938328), 4 (PV938318, PV938319, PV938320), 5 (PV938329, PV938330, PV938331), 6 (PV938321, PV938322, PV938323), 8 (PV938324, PV938325, PV938326), 9 (PV938332, PV938333, PV938334), 12 (PV938335, PV938336, PV938337). https://binary.pensoft.net/file/1456615This dataset is made available under the Open Database License (http://opendatacommons.org/licenses/odbl/1.0/). The Open Database License (ODbL) is a license agreement intended to allow users to freely share, modify, and use this Dataset while maintaining this same freedom for others, provided that the original source and author(s) are credited.Keyvan Esmaeilzadeh-Salestani, Mariana Bessa de Queiroz, Vladimir Mikryukov, Sylwia Uszok, Bruno Tomio Goto, Leho Tedersoo, Franco Magurno

Supplementary material 2 Phylogram generated from Maximum Likelihood analysis based on the eDNA dataset of Archaeosporales Data type pdf Explanation note The analysis conducted in IQ-TREE 2 involved 918 sequences, 563 of which overlapping the SSU-ITS-LSU region, representative of four families and seven genera in Archaeosporales. In Archaeosporaceae, candidate lineages at species rank are highlighted by colored boxes. Support values (SH-aLRT and Ultrafast Bootstrap support) are shown beside the respective lineage labels. Bar indicates 0.1 expected change per site per branch. https://binary.pensoft.net/file/1456616This dataset is made available under the Open Database License (http://opendatacommons.org/licenses/odbl/1.0/). The Open Database License (ODbL) is a license agreement intended to allow users to freely share, modify, and use this Dataset while maintaining this same freedom for others, provided that the original source and author(s) are credited.Keyvan Esmaeilzadeh-Salestani, Mariana Bessa de Queiroz, Vladimir Mikryukov, Sylwia Uszok, Bruno Tomio Goto, Leho Tedersoo, Franco Magurno

Supplementary material 3 ITS and LSU barcodes selected for the lineages identified in the study for each genus in Archaeosporaceae Data type pdf https://binary.pensoft.net/file/1456617This dataset is made available under the Open Database License (http://opendatacommons.org/licenses/odbl/1.0/). The Open Database License (ODbL) is a license agreement intended to allow users to freely share, modify, and use this Dataset while maintaining this same freedom for others, provided that the original source and author(s) are credited.Keyvan Esmaeilzadeh-Salestani, Mariana Bessa de Queiroz, Vladimir Mikryukov, Sylwia Uszok, Bruno Tomio Goto, Leho Tedersoo, Franco Magurno

Supplementary material 4 Global distribution of the genus *Antiquispora* based on EUKARYOME database records Data type pdf Explanation note The map shows the geographic locations (colored dots) where the genus has been recorded. A total of 596 sequences were considered from the reference database, spanning 65 countries (Poland for the type species, not included). Map generated with MapCustomizer (https://www.mapcustomizer.com/). https://binary.pensoft.net/file/1456618This dataset is made available under the Open Database License (http://opendatacommons.org/licenses/odbl/1.0/). The Open Database License (ODbL) is a license agreement intended to allow users to freely share, modify, and use this Dataset while maintaining this same freedom for others, provided that the original source and author(s) are credited.Keyvan Esmaeilzadeh-Salestani, Mariana Bessa de Queiroz, Vladimir Mikryukov, Sylwia Uszok, Bruno Tomio Goto, Leho Tedersoo, Franco Magurno

Supplementary material 5 Occurrence of fungal genera in the family Archaeosporaceae across different biomes, based on EUKARYOME database records Data type pdf Explanation note The table summarizes the presence of each genus in natural and anthropogenic biomes, including forests, grasslands, shrublands, woodlands, deserts, and aquatic environments. https://binary.pensoft.net/file/1456619This dataset is made available under the Open Database License (http://opendatacommons.org/licenses/odbl/1.0/). The Open Database License (ODbL) is a license agreement intended to allow users to freely share, modify, and use this Dataset while maintaining this same freedom for others, provided that the original source and author(s) are credited.Keyvan Esmaeilzadeh-Salestani, Mariana Bessa de Queiroz, Vladimir Mikryukov, Sylwia Uszok, Bruno Tomio Goto, Leho Tedersoo, Franco Magurno

Supplementary material 6 Global distribution of the genus *Andinospora* based on EUKARYOME database records Data type pdf Explanation note The map shows the geographic locations (colored dots) where the genus has been recorded. A total of 52 sequences from the reference database, spanning 14 countries (Ecuador for the type species, not included). Map generated with MapCustomizer (https://www.mapcustomizer.com/). https://binary.pensoft.net/file/1456620This dataset is made available under the Open Database License (http://opendatacommons.org/licenses/odbl/1.0/). The Open Database License (ODbL) is a license agreement intended to allow users to freely share, modify, and use this Dataset while maintaining this same freedom for others, provided that the original source and author(s) are credited.Keyvan Esmaeilzadeh-Salestani, Mariana Bessa de Queiroz, Vladimir Mikryukov, Sylwia Uszok, Bruno Tomio Goto, Leho Tedersoo, Franco Magurno

Supplementary material 7 Global distribution of the genus *Archaeospora* based on EUKARYOME database records Data type pdf Explanation note The map shows the geographic locations (colored dots) where the genus has been recorded. A total of 251 sequences were considered from the reference database, spanning 38 countries. Map generated with MapCustomizer (https://www.mapcustomizer.com/). https://binary.pensoft.net/file/1456621This dataset is made available under the Open Database License (http://opendatacommons.org/licenses/odbl/1.0/). The Open Database License (ODbL) is a license agreement intended to allow users to freely share, modify, and use this Dataset while maintaining this same freedom for others, provided that the original source and author(s) are credited.Keyvan Esmaeilzadeh-Salestani, Mariana Bessa de Queiroz, Vladimir Mikryukov, Sylwia Uszok, Bruno Tomio Goto, Leho Tedersoo, Franco Magurno

## References

[B1] Abarenkov K Nilsson RH Larsson KH Taylor AFS May TW Frøslev TG Pawlowska J Lindahl B Põldmaa K Truong C Vu D Hosoya T Niskanen T Piirmann T Ivanov F Zirk A Peterson M Cheeke TE Ishigami Y Jansson AT Jeppesen TS Kristiansson E Mikryukov V Miller JT Oono R Ossandon FJ Paupério J Saar I Schigel D Suija A Tedersoo L Kõljalg U (2024) The UNITE database for molecular identification and taxonomic communication of fungi and other eukaryotes: Sequences, taxa and classifications reconsidered. Nucleic Acids Research 5: 791–797. 10.1093/nar/gkad1039 PMC10767974 37953409

[B2] Ames RN Linderman RG (1976) *Acaulospora trappei* sp. nov. Mycotaxon 3: 565–569. 10.5962/p.414006

[B3] Barbera P Kozlov AM Czech L Morel B Darriba D Flouri T Stamatakis A (2019) EPA-ng: Massively Parallel Evolutionary Placement of Genetic Sequences. Systematic Biology 68(2): 365–369. 10.1093/sysbio/syy054 30165689 PMC6368480

[B4] Błaszkowski J (2012) Glomeromycota. W. Szafer Institute of Botany, Polish Academy of Sciences, Kraków, Poland.

[B5] Błaszkowski J Czerniawska B (2005) *Entrophospora schenckii* and *Pacispora franciscana*, arbuscular mycorrhizal fungi (Glomeromycota) new for Europe and Poland, respectively. Acta Mycologica 40(1): 11–18. 10.5586/am.2005.002

[B6] Błaszkowski J Niezgoda P Meller E Milczarski P Zubek S Malicka M Uszok S Casieri L Goto BT Magurno F (2021) New taxa in Glomeromycota: Polonosporaceae fam. nov., *Polonospora* gen. nov., and *P. polonica* comb. nov. Mycological Progress 20: 941–951. 10.1007/s11557-021-01726-4

[B7] Błaszkowski J Sánchez-García M Niezgoda P Zubek S Fernández F Vila A Al-Yahya’ei MN Symanczik S Milczarski P Malinowski R Cabello M Goto BT Casieri L Malicka M Bierza W Magurno F (2022) A new order, Entrophosporales, and three new *Entrophospora* species in Glomeromycota. Frontiers in Microbiology 13: 962856. 10.3389/fmicb.2022.962856 PMC9835108 36643412

[B8] Błaszkowski J Zubek S Milczarski P Malinowski R Niezgoda P Goto BT (2025a) New taxa and a combination in Glomerales (Glomeromycota, Glomeromycetes). MycoKeys 112: 253–276. 10.3897/mycokeys.112.136158 39886477 PMC11780324

[B9] Błaszkowski J Goto BT Zubek S Milczarski P Malinowski R Niezgoda P Błaszkowski T (2025b) *Paracorymbiglomus* gen. nov., *Diversispora conica* sp. nov., and new combinations in Diversisporaceae (Glomeromycota). MycoKeys 117: 171–190. 10.3897/mycokeys.117.148052 40364894 PMC12070055

[B10] Brundrett MC Tedersoo L (2018) Evolutionary history of mycorrhizal symbioses and global host plant diversity. The New Phytologist 220: 1108–1115. 10.1111/nph.14976 29355963

[B11] Corradi N Antunes PM Magurno F (2025) A call for reform: Implementing genome-based approaches for species classification in Glomeromycotina. The New Phytologist 247(1): 50–54. 10.1111/nph.70148 40235302

[B12] Cowgill C Gilbert JD Convery I Handley L (2025) Monitoring terrestrial rewilding with environmental DNA metabarcoding: A systematic review of current trends and recommendations. Frontiers in Conservation Science 5: 1473957. 10.3389/fcosc.2024.1473957

[B13] da Silva GA de Assis DMA Sieverding E Oehl F (2024) Four New Families of Arbuscular Mycorrhizal Fungi Within the Order Glomerales. Taxonomy 4: 761–779. 10.3390/taxonomy4040041

[B14] da Silva GA Sieverding E Assis DMA Goto BT Corazon-Guivin MA Oehl F (2025) Revision of Entrophosporales, with Three Genera and an Identification Key for All Species Currently Attributed to This Order. Journal of Fungi 11: 97. 10.3390/jof11020097 PMC11856536 39997391

[B15] Duarte S Vieira PE Lavrador AS Costa FO (2021) Status and prospects of marine NIS detection and monitoring through (e) DNA metabarcoding. The Science of the Total Environment 751: 141729. 10.1016/j.scitotenv.2020.141729 32889465

[B16] Fall AF Nakabonge G Ssekandi J Founoune-Mboup H Apori SO Ndiaye A Badji A Ngom K (2022) Roles of Arbuscular Mycorrhizal Fungi on Soil Fertility: Contribution in the Improvement of Physical, Chemical, and Biological Properties of the Soil. Frontiers in Fungal Biology 3: 723892. 10.3389/ffunb.2022.723892 PMC10512336 37746193

[B17] Fediajevaite J Priestley V Arnold R Savolainen V (2021) Meta-analysis shows that environmental DNA outperforms traditional surveys, but warrants better reporting standards. Ecology and Evolution 11: 4803–4815. 10.1002/ece3.7382 33976849 PMC8093654

[B18] Goto BT Queiroz MB Magurno F Souza FA Błaszkowski J (2024) How far have we progressed in Glomeromycota taxonomy and systematic? IMS newsletter 5: 18–24.

[B19] Hafeel KM (2004) Spore ontogeny of the arbuscular mycorrhizal fungus *Archaeospora trappei* (Ames & Schneider) Morton & Redecker (Archaeosporaceae). Mycorrhiza 14: 213–219. 10.1007/s00572-004-0300-y 14991467

[B20] Hug LA Baker BJ Anantharaman K Brown CT Probst AJ Castelle CJ Butterfield CN Hernsdorf AW Amano Y Ise K Suzuki Y Dudek N Relman DA Finstad KM Amundson R Thomas BC Banfield JF (2016) A new view of the tree of life. Nature Microbiology 1: 16048. 10.1038/nmicrobiol.2016.48 27572647

[B21] Hyde KD (1995) Fungi from palms. XXVIII. *Appendicospora coryphae*, a new name for Apiosporella coryphae. Sydowia 47: 31–37.

[B22] Katoh K Standley DM (2013) MAFFT multiple sequence alignment software version 7: Improvements in performance and usability. Molecular Biology and Evolution 30: 772–780. 10.1093/molbev/mst010 23329690 PMC3603318

[B23] Kestel JH Field DL Bateman PW White NE Allentoft ME Hopkins AJM Gibberd M Nevill P (2022) Applications of environmental DNA (eDNA) in agricultural systems: Current uses, limitations and future prospects. The Science of the Total Environment 847: 157556. 10.1016/j.scitotenv.2022.157556 35882340

[B24] Kornerup A Wanscher JH (1983) Methuen handbook of colour, 3^rd^ edn. Eyre Methuen, London, 252 pp.

[B25] Kozlov AM Darriba D Flouri T Morel B Stamatakis A (2019) RAxML-NG: A fast, scalable, and user-friendly tool for maximum likelihood phylogenetic inference. Bioinformatics 35(21): 4453–4455. 10.1093/bioinformatics/btz305 31070718 PMC6821337

[B26] Krüger M Stockinger H Krüger C Schüßler A (2009) DNA-based species level detection of Glomeromycota: One PCR primer set for all arbuscular mycorrhizal fungi. The New Phytologist 183(1): 212–223. 10.1111/j.1469-8137.2009.02835.x 19368665

[B27] Larsson A (2014) AliView: A fast and lightweight alignment viewer and editor for large datasets. Bioinformatics 30: 3276–3278. 10.1093/bioinformatics/btu531 25095880 PMC4221126

[B28] Magurno F Uszok S Bierza K Bakr J Kende Z Queiroz MB Casieri L (2024) *Glomus mongioiense*, a New Species of Arbuscular Mycorrhizal Fungi from Italian Alps and the Phylogeny-Spoiling Issue of Ribosomal Variants in the *Glomus* Genus. Agronomy 14: 1350. 10.3390/agronomy14071350

[B29] Malicka M Magurno F Piotrowska-Seget Z (2022) Phenol and polyaromatic hydrocarbons are stronger drivers than host plant species in shaping the arbuscular mycorrhizal fungal component of the mycorrhizosphere. International Journal of Molecular Sciences 23: 12585. 10.3390/ijms232012585 PMC9604154 36293448

[B30] Miller MA Pfeiffer W Schwartz T (2010) Creating the CIPRES science gateway for inference of large phylogenetic trees. Proceedings of the Gateway Computing Environments Workshop, (USA), New Orleans, LA, 14: 1–8. 10.1109/GCE.2010.5676129

[B31] Minh BQ Schmidt HA Chernomor O Schrempf D Woodhams MD Von Haeseler A Lanfear R (2020) IQ-TREE 2: New models and efficient methods for phylogenetic inference in the Genomic era. Molecular Biology and Evolution 37: 1530–1534. 10.1093/molbev/msaa015 32011700 PMC7182206

[B32] Morton JB Redecker D (2001) Two new families of Glomales, Archaeosporaceae and Paraglomaceae, with two new genera *Archaeospora* and *Paraglomus*, based on concordant molecular and morphological characters. Mycologia 93(1): 181–195. 10.1080/00275514.2001.1206314

[B33] Oehl F Silva G Goto BT Sieverding E (2011) New recombinations in Glomeromycota. Mycotaxon 117: 429–434. 10.5248/117.429

[B34] Oehl F Sánchez-Castro I Palenzuela J da Silva GA (2015) *Palaeospora spainii*, a new arbuscular mycorrhizal fungus from Swiss agricultural soils. Nova Hedwigia 101(1): 89–102. 10.1127/nova_hedwigia/2014/02

[B35] Rambaut A (2018) FigTree v1.4.4. https://github.com/rambaut/figtree/releases

[B36] Ronquist F Teslenko M van der Mark P Ayres DL Darling A Höhna S Larget B Liu L Suchard MA Huelsenbeck JP (2012) MrBayes 3.2: Efficient Bayesian phylogenetic inference and model choice across a large model space. Systematic Biology 61(3): 539–542. 10.1093/sysbio/sys029 22357727 PMC3329765

[B37] Sayers EW Cavanaugh M Clark K Pruitt KD Sherry ST Yankie L Karsch-Mizrachi I (2024) GenBank 2024 Update. Nucleic Acids Research 52: 134–137. 10.1093/nar/gkad903 PMC10767886 37889039

[B38] Schüßler A Walker C (2010) The Glomeromycota: A Species List with New Families and New Genera. Royal Botanic Garden Edinburgh.

[B39] Schüßler A Walker C (2019) *Archaeospora ecuadoriana* sp. nov. from a mountainous biodiversity hotspot area in Ecuador, and transfer of *Palaeospora spainiae* to *Archaeospora*, as *A. spainiae* comb. nov. Mycorrhiza 29: 435–443. 10.1007/s00572-019-00913-2 31446486

[B40] Schüßler A Schwarzott D Walker C (2001) A new fungal phylum, the Glomeromycota: Evolution and phylogeny. Mycological Research 105: 1413–1421. 10.1017/S0953756201005196

[B41] Sieverding E Oehl F (2006) Revision of *Entrophospora* and description of *Kuklospora* and *Intraspora*, two new genera in the arbuscular mycorrhizal Glomeromycetes. Journal of Applied Botany and Food Quality 80: 69–81.

[B42] Spain JL (2003) Emendation of *Archaeospora* and of its type species, *Archaeospora trappei*. Mycotaxon 87: 109–112.

[B43] Spain J Oehl F Sieverding E (2006) *Appendicispora* a new genus in the arbuscular mycorrhiza-forming Glomeromycetes, with a discussion of the genus *Archaeospora*. Mycotaxon 97: 163–182.

[B44] Steenwyk JL Buida TJ Li Y Shen X-X Rokas A (2020) ClipKIT: A multiple sequence alignment trimming software for accurate phylogenomic inference. PLoS Biology 18: e3001007. 10.1371/journal.pbio.3001007 PMC7735675 33264284

[B45] Stöver BC Müller KF (2010) TreeGraph 2: Combining and visualizing evidence from different phylogenetic analyses. BMC Bioinformatics 11(7): 1–9. 10.1186/1471-2105-11-7 20051126 PMC2806359

[B46] Tedersoo L Magurno F Alkahtani S Mikryukov V (2024a) Phylogenetic classification of arbuscular mycorrhizal fungi: New species and higher-ranking taxa in Glomeromycota and Mucoromycota (class Endogonomycetes). MycoKeys 107: 249–271. 10.3897/mycokeys.107.125549 39169987 PMC11336396

[B47] Tedersoo L Hosseyni Moghaddam MS Mikryukov V Hakimzadeh A Bahram M Nilsson RH Yatsiuk I Geisen S Schwelm A Piwosz K Prous M Chmolowska D Rueckert S Skaloud P Laas P Thines M Jung J-H Alkahtani S Anslan S (2024b) EUKARYOME: The rRNA gene reference database for identification of all eukaryotes. Database : The Journal of Biological Databases and Curation 2024: baae043. 10.1093/database/baae043 PMC11168333 38865431

[B48] Walker C (2008) *Ambispora* and Ambisporaceae resurrected. Mycological Research 112: 297–298. 10.1016/j.mycres.2008.02.001

[B49] Walker C Vestberg M Demircik F Stockinger H Saito M Sawaki H Nishmura I Schüssler A (2007a) Molecular phylogeny and new taxa in the Archaeosporales (Glomeromycota): *Ambispora fennica* gen. sp. nov., Ambisporaceae fam. nov., and emendation of *Archaeospora* and Archaeosporaceae. Mycological Research 111(2): 137–153. 10.1016/j.mycres.2006.11.008 17324754

[B50] Walker C Vestberg M Schüssler A (2007b) Nomenclatural clarifications in Glomeromycota. Mycological Research 111: 253–255. 10.1016/j.mycres.2007.02.009 17324754

