# Corrigendum: Esmaeilzadeh-Salestani K, Queiroz MB, Mikryukov V, Uszok S, Goto BT, Tedersoo L, Magurno F (2025) Morphological and phylogenetic analysis of the early-diverging lineage of Glomeromycota suggest two new genera and recombinations in Archaeosporales. MycoKeys 124: 249-273. https://doi.org/10.3897/mycokeys.124.166449

**DOI:** 10.3897/mycokeys.127.182916

**Published:** 2026-01-29

**Authors:** Bruno Tomio Goto, Mariana Bessa de Queiroz, Keyvan Esmaeilzadeh-Salestani, Vladimir Mikryukov, Sylwia Uszok, Leho Tedersoo, Franco Magurno

**Affiliations:** 1 Departamento de Botânica e Zoologia, Universidade Federal do Rio Grande do Norte, Campus Universitário, Natal 59072-970, RN, Brazil Faculty of Natural Sciences, University of Silesia in Katowice Katowice Poland; 2 Programa de Pós-graduação em Sistemática e Evolução, Centro de Biociências, Universidade Federal do Rio Grande do Norte, Campus Universitário, Natal 59072-970, RN, Brazil College of Science, King Saud University Riyadh Saudi Arabia; 3 Mycology and Microbiology Center, University of Tartu, Liivi 2, 50409 Tartu, Estonia Institute of Technology, University of Tartu Tartu Estonia; 4 Institute of Technology, University of Tartu, Nooruse 1, 50411 Tartu, Estonia Mycology and Microbiology Center, University of Tartu Tartu Estonia; 5 Institute of Ecology and Earth Sciences, University of Tartu, Liivi 2, 50409 Tartu, Estonia Institute of Ecology and Earth Sciences, University of Tartu Tartu Estonia; 6 Institute of Biology, Biotechnology and Environmental Protection, Faculty of Natural Sciences, University of Silesia in Katowice, Jagiellońska 28, 40-032, Katowice, Poland Departamento de Botânica e Zoologia, Universidade Federal do Rio Grande do Norte Natal Brazil; 7 Department of Zoology, College of Science, King Saud University, 12371 Riyadh, Saudi Arabia Centro de Biociências, Universidade Federal do Rio Grande do Norte Natal Brazil

After reviewing the paper recently published by Esmaeilzadeh-Salestani et al. (2025), we noticed that the names *Andinospora* and *Andinospora ecuadoriana*, as well as the *Archaeospora* emendation, are to be considered formally invalid due to the use of the term “type genus” instead of “type species” when referring to *Andinospora ecuadoriana* and *Archaeospora trappei*, as required by Art. 40.1 and 41.5 (Turland et al. 2018). Therefore, the sentences containing the inappropriate term have been amended as follows.

## Andinospora

Taxon classificationFungiArchaeosporales

Magurno, Uszok, Esmaeilzadeh-Salestani, Tedersoo, M.B. Queiroz & B.T. Goto gen. nov.

F6735C3D-F6E7-5976-BDA1-93AC068E79B5

860983

### Type species.

*Andinospora ecuadoriana* (A. Schüßler & C. Walker) Magurno, Uszok, M.B. Queiroz & B.T. Goto, comb. nov. MB 860984.

### Basionym.

*Archaeospora ecuadoriana* A. Schüßler & C. Walker, Mycorrhiza 29: 437 (2019) MB 556466.

## Archaeospora

Taxon classificationFungiArchaeosporalesArchaeosporaceae

(J.B. Morton & D. Redecker) emend. Magurno, Uszok, Esmaeilzadeh-Salestani, Tedersoo, M.B. Queiroz & B.T. Goto

29C14B4E-C625-5F1B-9220-07E3FCBBC776

### Type species.

*Archaeospora trappei* (R.N. Ames & Linderman) J.B. Morton & D. Redecker, Mycologia 93 (1): 183 (2001) MB 467737.

### Basionym.

*Acaulospora trappei* R.N. Ames & Linderman, Mycotaxon 3 (3): 566 (1976) MB 308080.

## Supplementary Material

XML Treatment for Andinospora

XML Treatment for Archaeospora

## Additional information

### Use of AI

No use of AI was reported.

### Author ORCIDs

Bruno Tomio Goto https://orcid.org/0000-0001-6157-4954

Mariana Bessa de Queiroz https://orcid.org/0000-0003-0197-7203

Keyvan Esmaeilzadeh-Salestani https://orcid.org/0000-0002-6882-7616

Vladimir Mikryukov https://orcid.org/0000-0003-2786-2690

Sylwia Uszok https://orcid.org/0000-0001-5961-7695

Leho Tedersoo https://orcid.org/0000-0002-1635-1249

Franco Magurno https://orcid.org/0000-0002-3117-8149

### Data availability

All of the data that support the findings of this study are available in the main text.
