# Supplementary figures and images for: ﻿Morphological and phylogenetic analysis of the early-diverging lineage of Glomeromycota suggest two new genera and recombinations in Archaeosporales
Source: MycoKeys. 2025 Nov 3;124:249–73. doi: 10.3897/mycokeys.124.166449 (PMC12603645; doi:10.3897/mycokeys.124.166449)

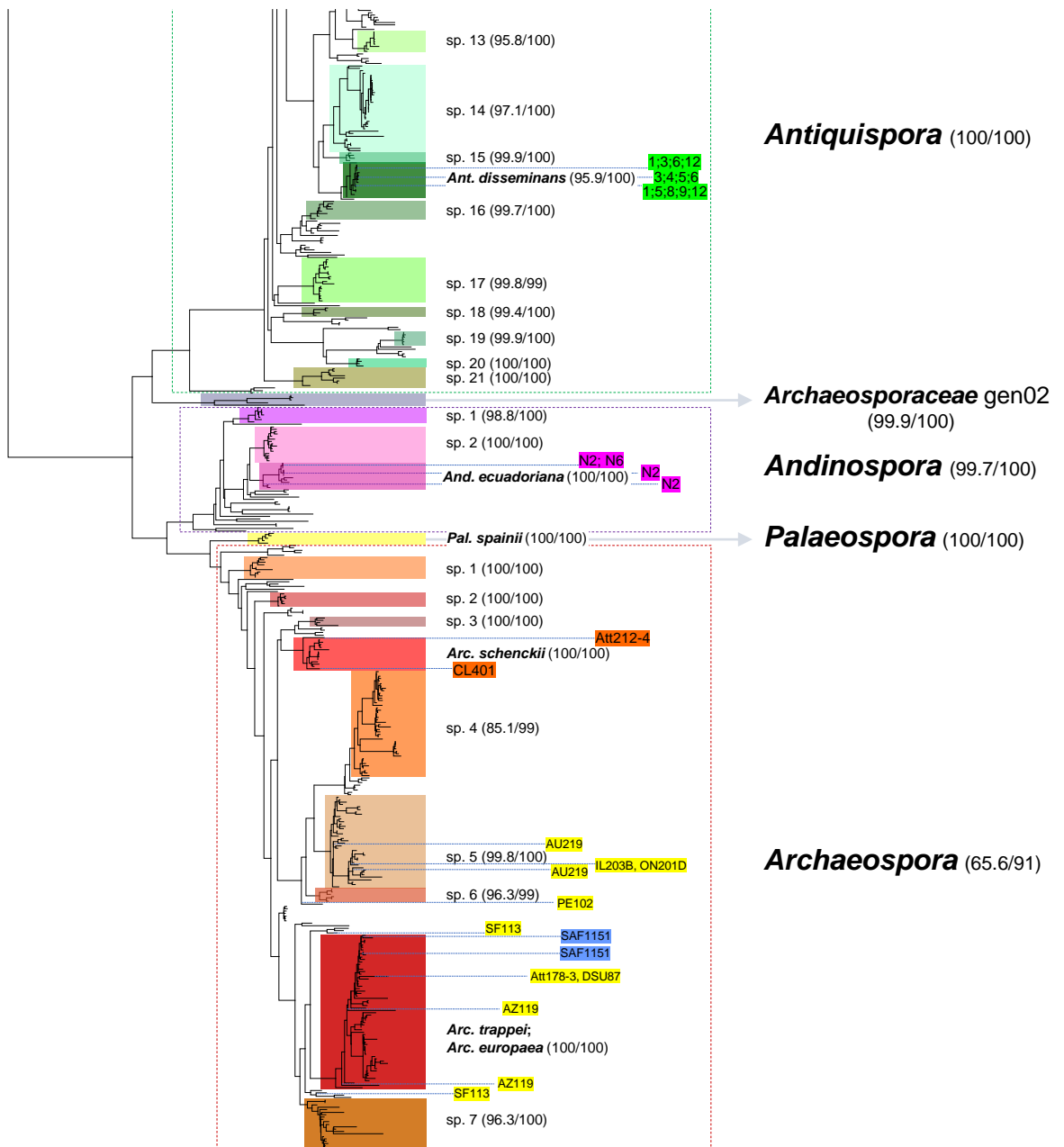

Supplement: Supplementary material 1 — Snapshot of EPA analysis to infer the position of Archaeospora trappei, Arc. schenckii, Arc. europaea, Andinospora ecuadoriana and Antiquispora disseminans isolates in the reference eDNA tree [file mycokeys-124-249-s001.pdf]

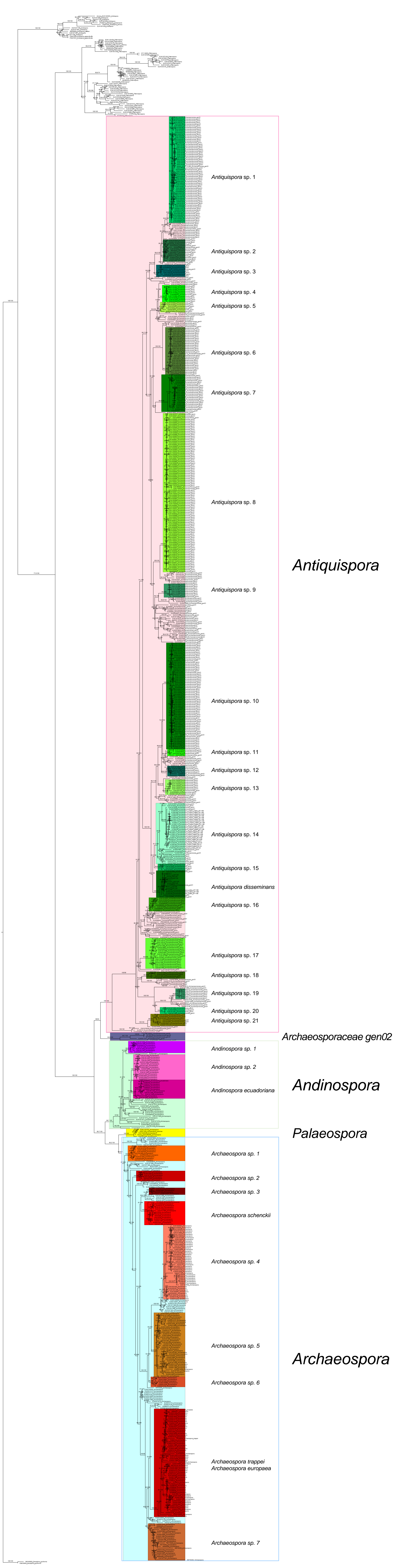

Supplement: Supplementary material 2 — Phylogram generated from Maximum Likelihood analysis based on the eDNA dataset of Archaeosporales [file mycokeys-124-249-s002.pdf]

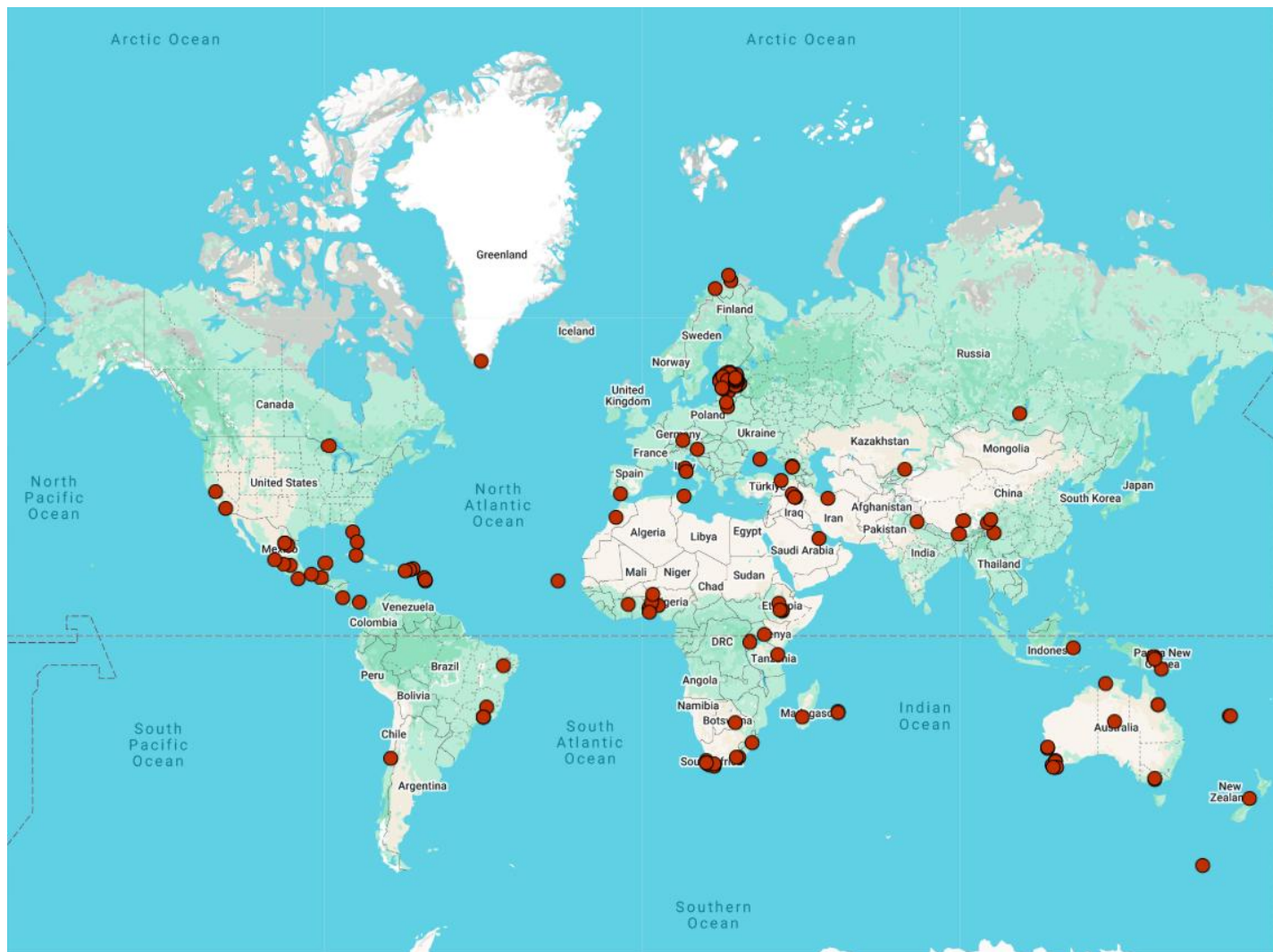

Supplement: Supplementary material 4 — Global distribution of the genus Antiquispora based on EUKARYOME database records [file mycokeys-124-249-s004.pdf]

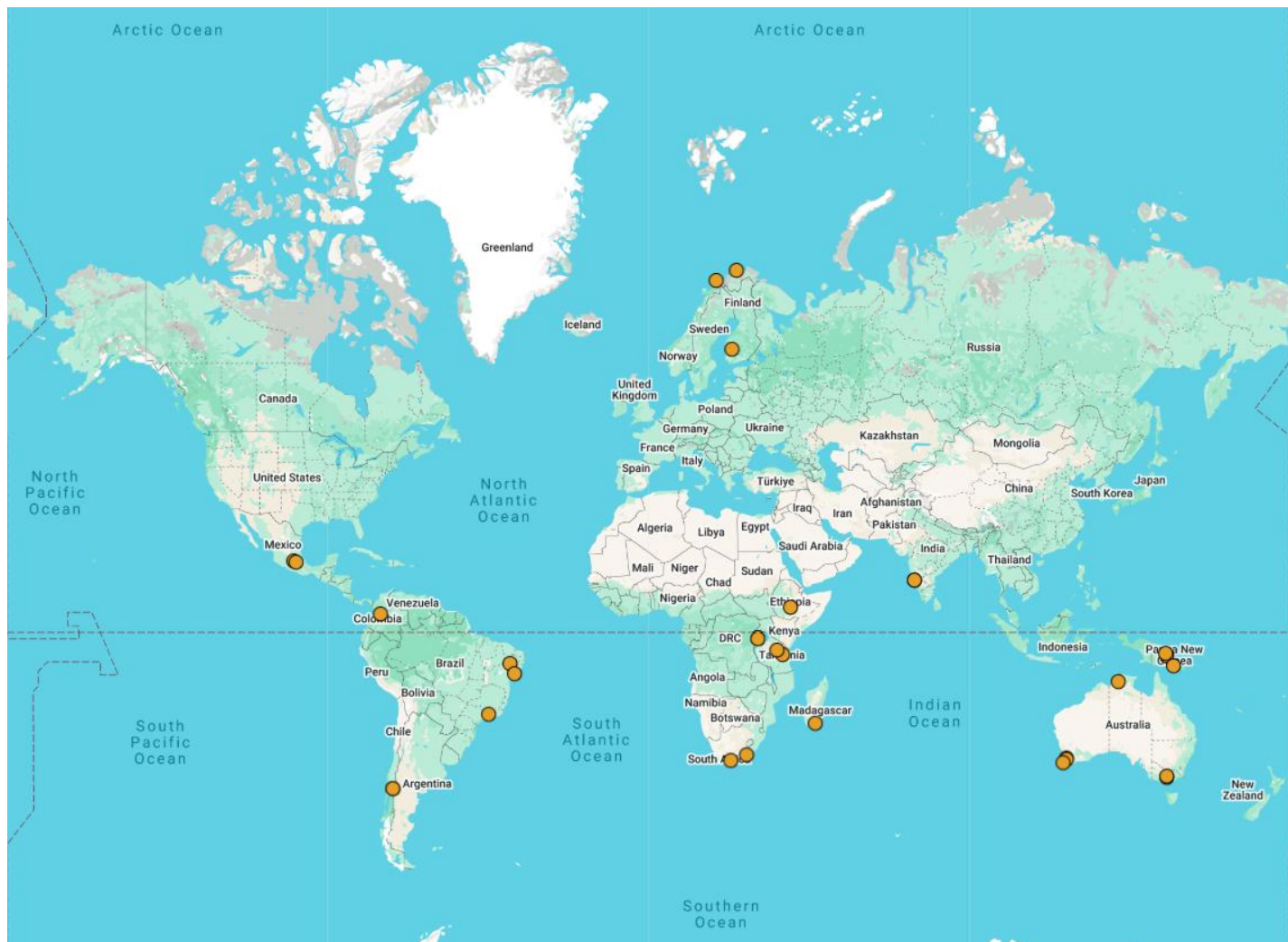

Supplement: Supplementary material 6 — Global distribution of the genus Andinospora based on EUKARYOME database records [file mycokeys-124-249-s006.pdf]

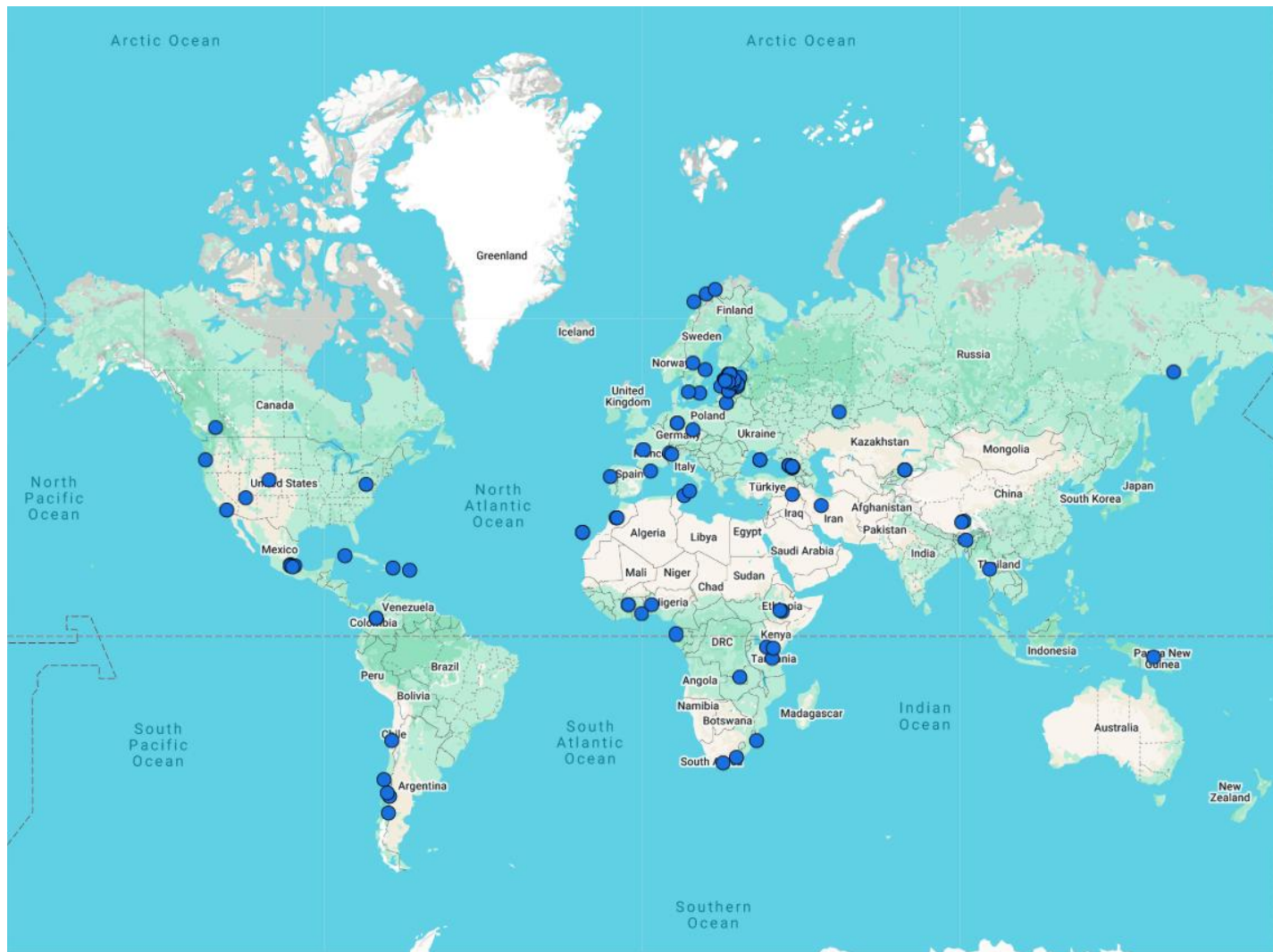

Supplement: Supplementary material 7 — Global distribution of the genus Archaeospora based on EUKARYOME database records [file mycokeys-124-249-s007.pdf]
