# Supplementary material for: ﻿Morphological and phylogenetic analysis of the early-diverging lineage of Glomeromycota suggest two new genera and recombinations in Archaeosporales
Source: MycoKeys. 2025 Nov 3;124:249–73. doi: 10.3897/mycokeys.124.166449 (PMC12603645; doi:10.3897/mycokeys.124.166449)
Supplement: Supplementary material 3 — ITS and LSU barcodes selected for the lineages identified in the study for each genus in Archaeosporaceae [file mycokeys-124-249-s003.pdf]

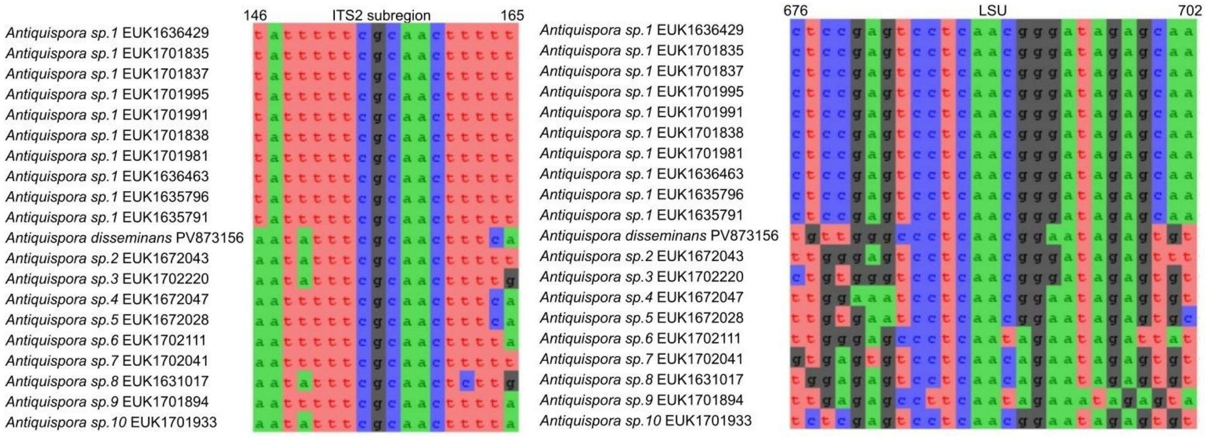

Figure S1. Separation of *Antiquispora sp.1* from other species of *Antiquispora* based on ITS region (ITS2 positions 146–165 tatttttcgcaactttttgg; no mismatch allowed) and LSU (positions 676–702 ctccgagtcctcaacgggatagagcaa; one mismatch allowed).

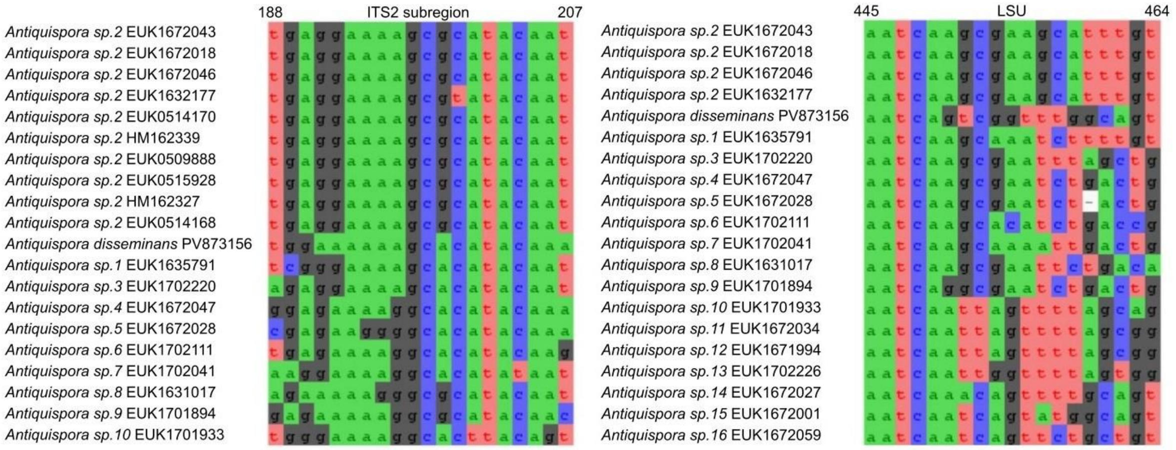

Figure S2. Separation of *Antiquispora sp.2* from other species of *Antiquispora* based on the ITS region (ITS2 positions 188–207 tgaggaaaagcgcatacaat; one mismatch allowed) and LSU (positions 445–464 aatcaagcgaagcatttgtc; no mismatch allowed).

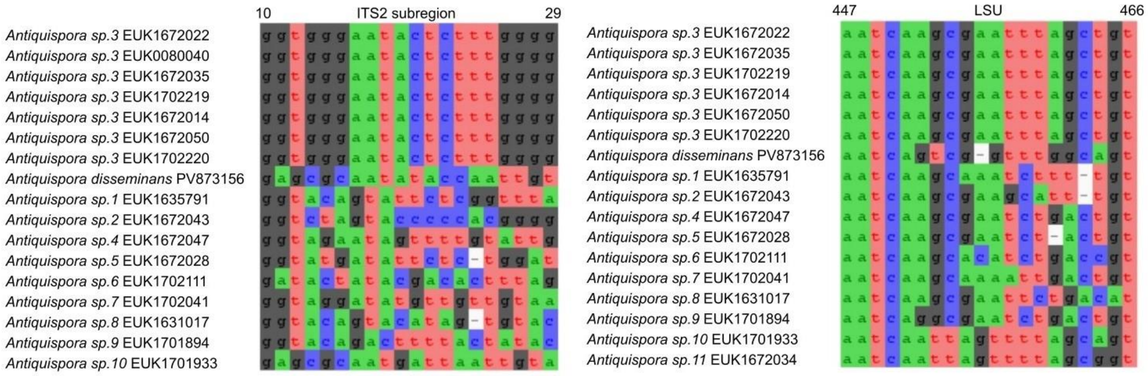

Figure S3. Separation of *Antiquispora sp.3* from other species of *Antiquispora* based on the ITS region (ITS2 positions 10–29 ggtgggaatactctttgggg; no mismatch allowed) and LSU (positions 447–466 aatcaagcgaatttagctgt; no mismatch allowed).

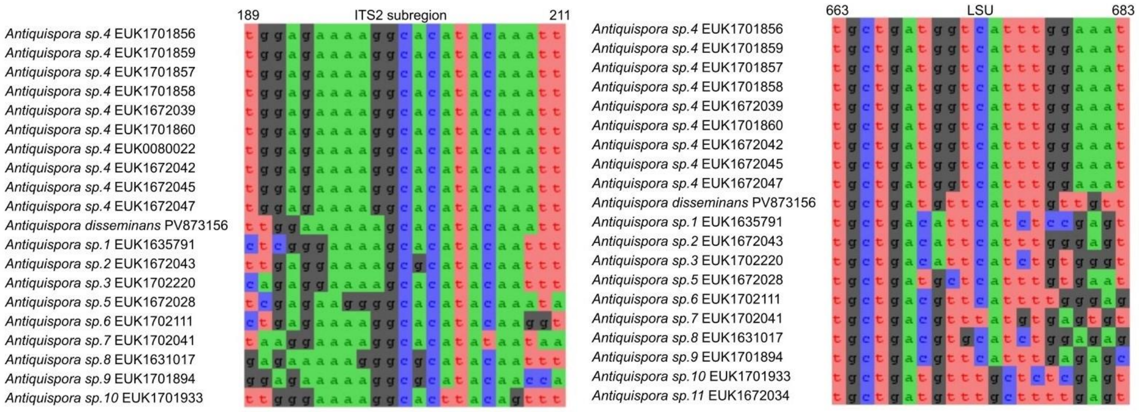

Figure S4. Separation of *Antiquispora sp.4* from other species of *Antiquispora* based on the ITS region (ITS2 positions 189–211 tggagaaaaggcacatacaaatt; no mismatch allowed) and LSU (positions 663–683 tgctgatggtcatttggaaat; no mismatch allowed).

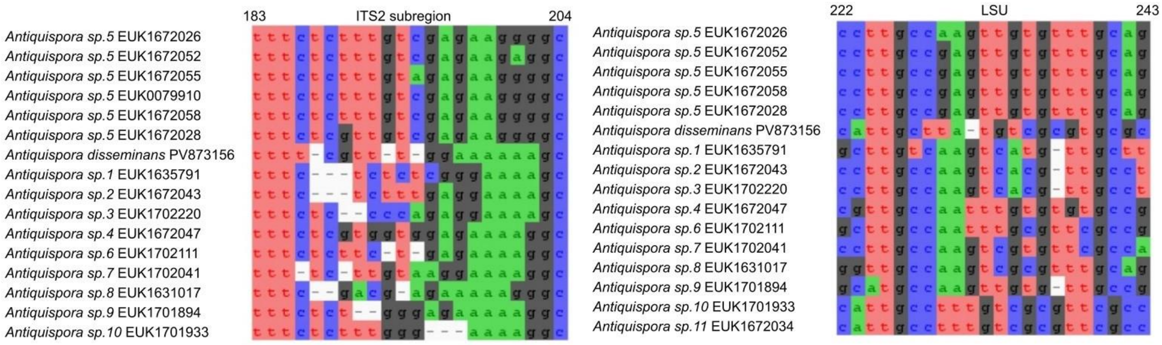

Figure S5. Separation of *Antiquispora sp.5* from other species of *Antiquispora* based on the ITS region (ITS2 positions 183–204 tttctctttgtcgagaaggggc; one mismatch allowed) and LSU (positions 222–243 ccttgccgagttgtgtttgcag; one mismatch allowed).

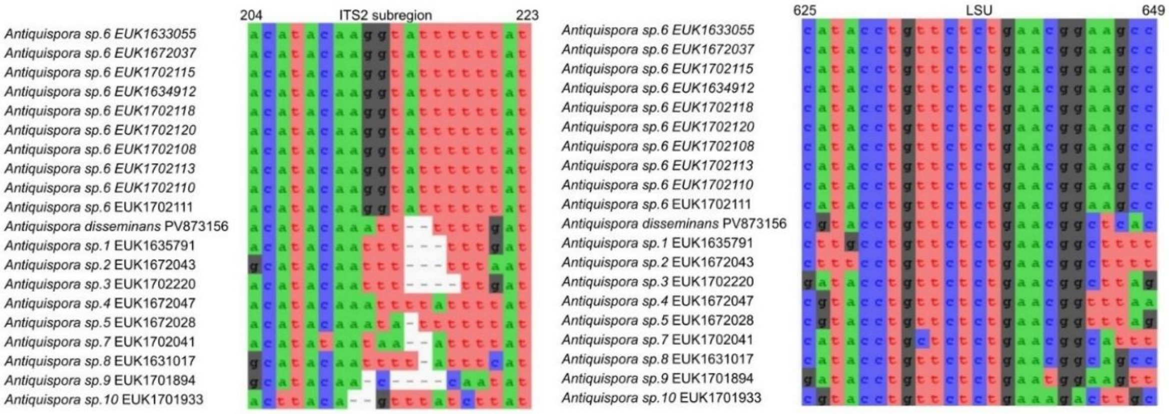

Figure S6. Separation of *Antiquispora sp.6* from other species of *Antiquispora* based on the ITS region (ITS2 positions 204–223 acatacaaggtattttttat; no mismatch allowed) and LSU (positions 625–649 catacctgttctctgaacggaagcc; no mismatch allowed).

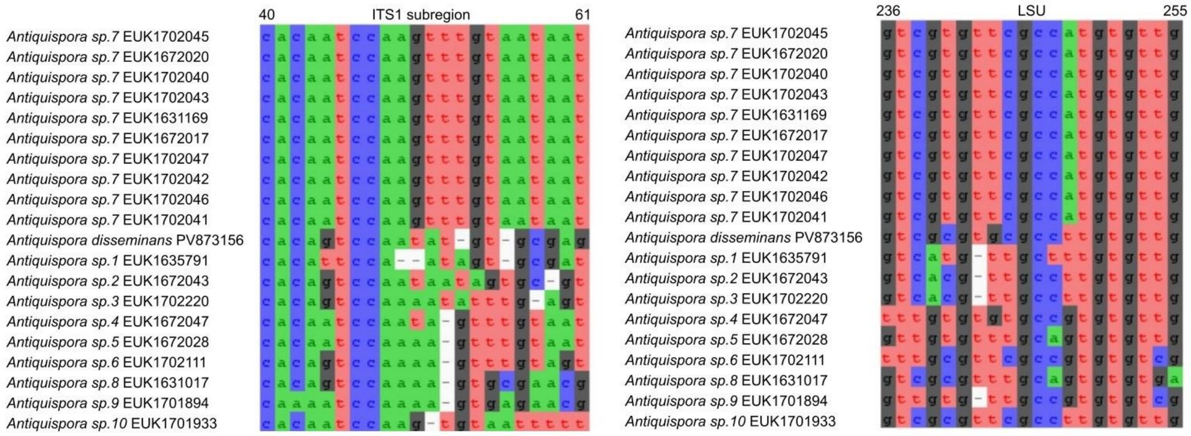

Figure S7. Separation of *Antiquispora sp.7* from other species of *Antiquispora* based on the ITS region (ITS1 positions 40–61 cacaatccaagtttgtaataat; one mismatch allowed) and LSU (positions 236–255 gtcgtgttcgccatgtgttg; no mismatch allowed).

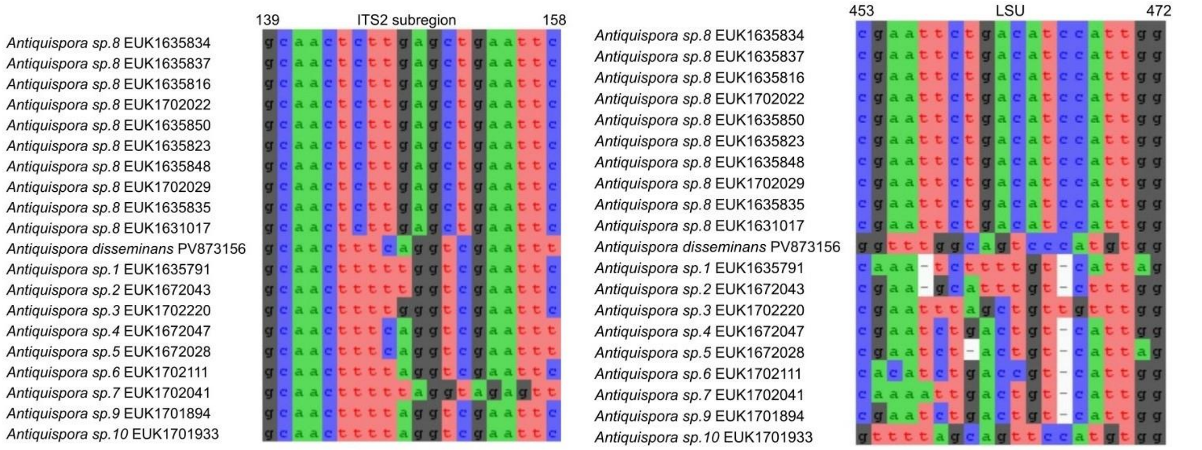

Figure S8. Separation of *Antiquispora sp.8* from other species of *Antiquispora* based on the ITS region (ITS2 positions 139–158 gcaactcttgagctgaattc; one mismatch allowed) and LSU (positions 453-472 cgaattctgacatccattgg; no mismatch allowed).

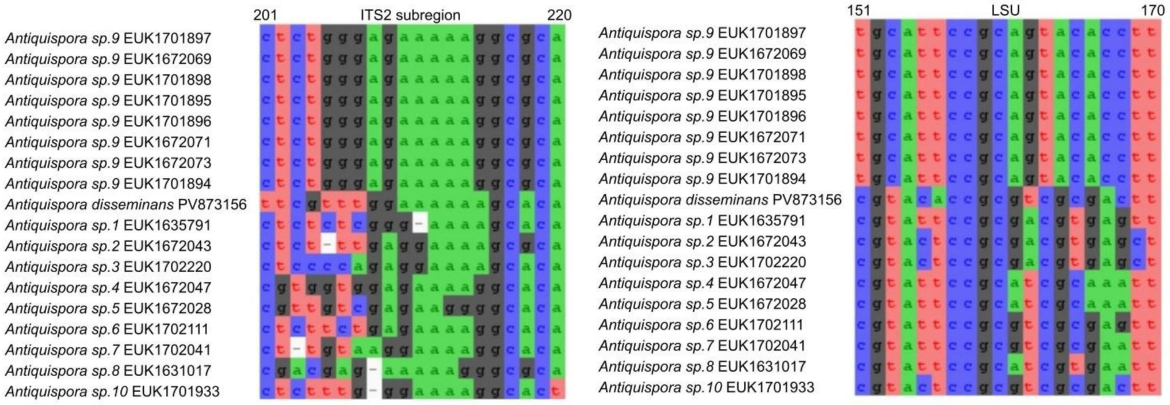

Figure S9. Separation of *Antiquispora sp.9* from other species of *Antiquispora* based on the ITS region (ITS2 positions 201–220 ctctgggagaaaaaggcgca; no mismatch allowed) and LSU (positions 151–170 tgcattccgcagtacacctt; no mismatch allowed).

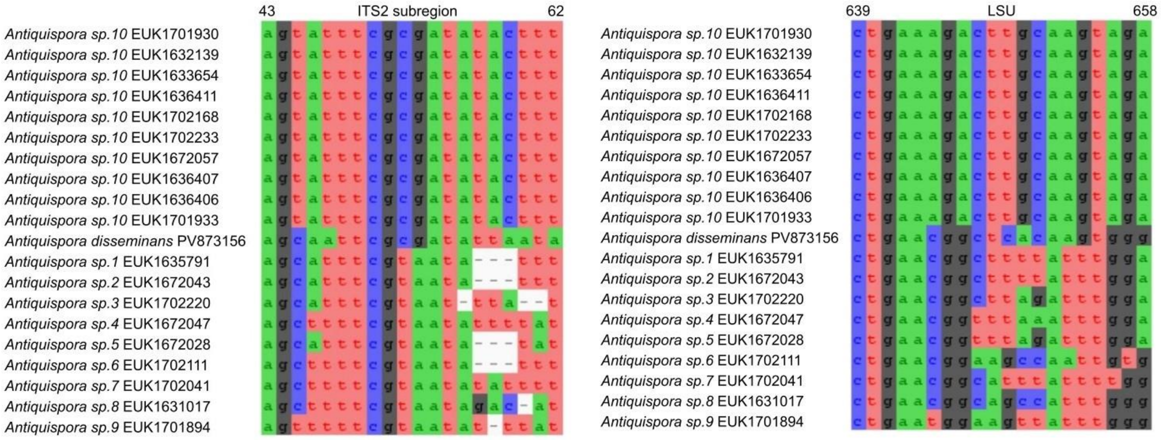

Figure S10. Separation of *Antiquispora sp.10* from other species of *Antiquispora* based on the ITS region (ITS2 positions 43–62 agtatttcgcgatatacttt; no mismatch allowed) and LSU (positions 639–658 ctgaaagacttgcaagtaga; one mismatch allowed).

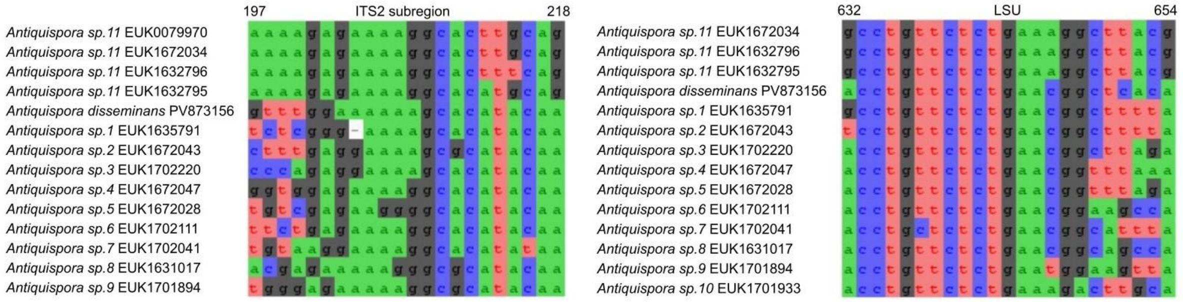

Figure S11. Separation of *Antiquispora sp.11* from other species of *Antiquispora* based on the ITS region (ITS2 positions 197–218 aaaagagaaaaggcacttgcag; one mismatch allowed) and LSU (positions 632–654 gcctgttctctgaaaggcttacg; one mismatch allowed).

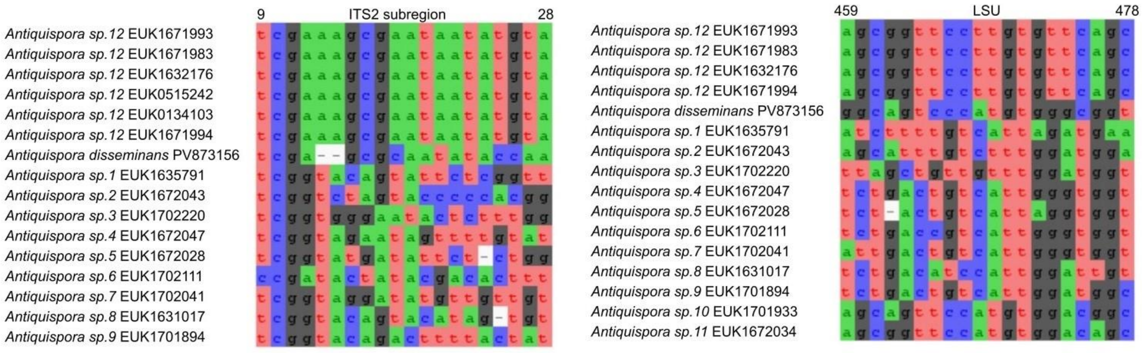

Figure S12. Separation of *Antiquispora sp.12* from other species of *Antiquispora* based on the ITS region (ITS2 positions 9–28 tcgaaagcgaataatatgta; no mismatch allowed) and LSU (positions 459–478 agcggttccttgtgttcagc; one mismatch allowed).

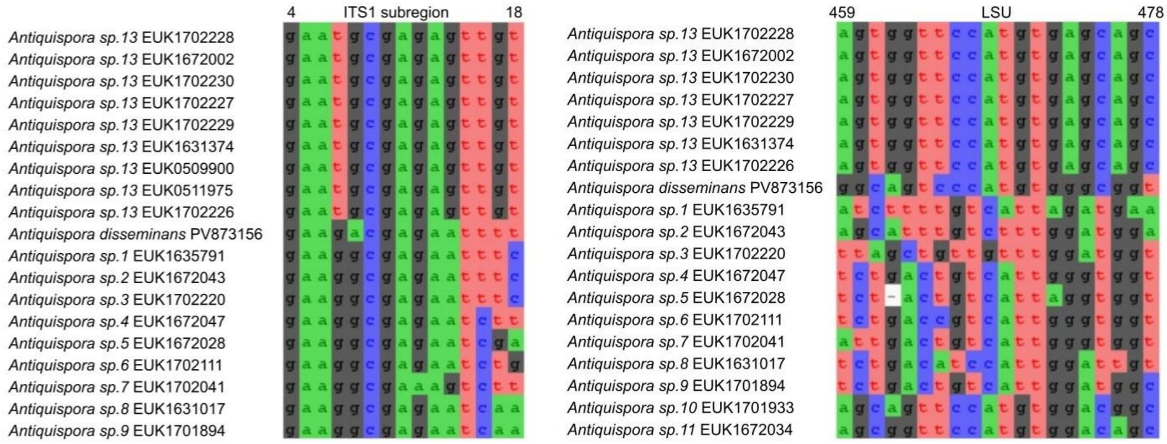

Figure S13. Separation of *Antiquispora sp.13* from other species of *Antiquispora* based on ITS region (ITS1 positions 4–18 gaatgcgagagttgt; no mismatch allowed) and LSU (positions 459–478 agtggttccatgtgagcagc; no mismatch allowed).

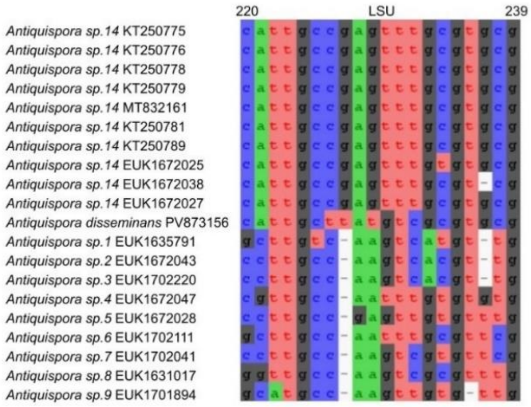

Figure S14. Separation of *Antiquispora sp.14* from other species of *Antiquispora* based on the LSU (positions 220–239 cattgccgagtttgcgtgcg; one mismatch allowed).

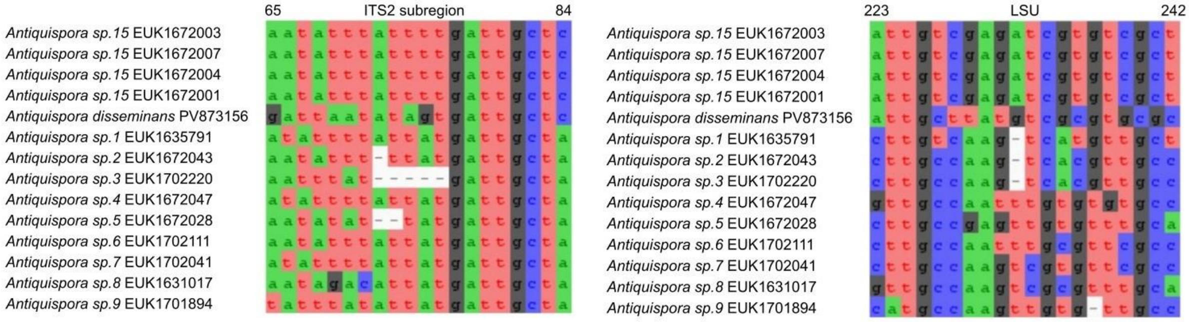

Figure S15. Separation of *Antiquispora sp.15* from other species of *Antiquispora* based on the ITS region (ITS2 positions 65–84 aatatttattttgattgctc; no mismatch allowed) and LSU (positions 223–242 attgtcgagatcgtgtcgct; no mismatch allowed).

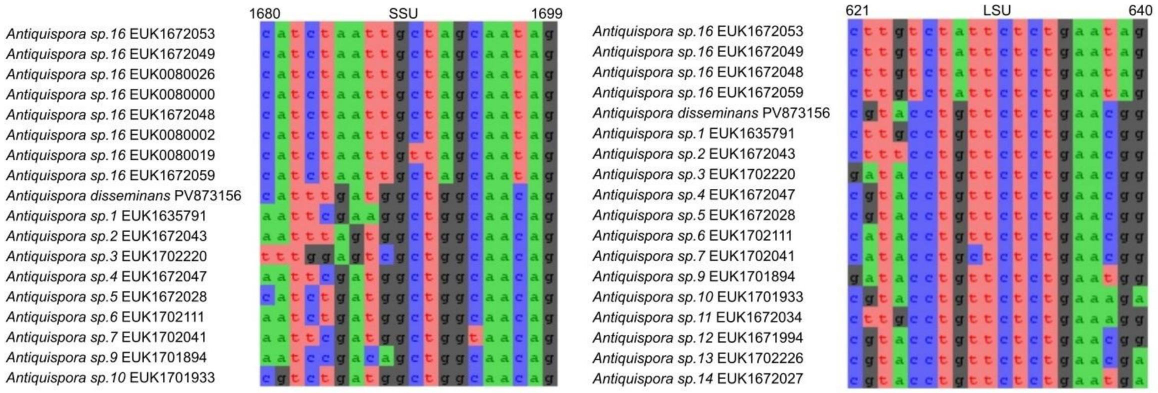

Figure S16. Separation of *Antiquispora sp.16* from other species of *Antiquispora* based on the SSU region (positions 1680–1699 catctaattgctagcaatag; one mismatch allowed) and LSU (positions 621–640 cttgtctattctctgaatag; no mismatch allowed).

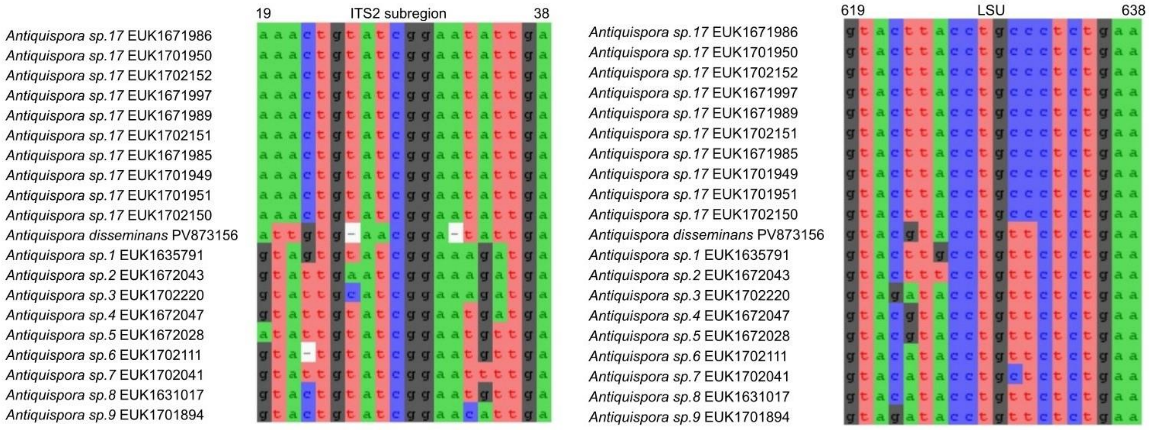

Figure S17. Separation of *Antiquispora sp.17* from other species of *Antiquispora* based on the ITS region (ITS2 positions 19–38 aaactgtatcggaatattga; no mismatch allowed) and LSU (positions 619–638 gtacttacctgccctctgaa; no mismatch allowed).

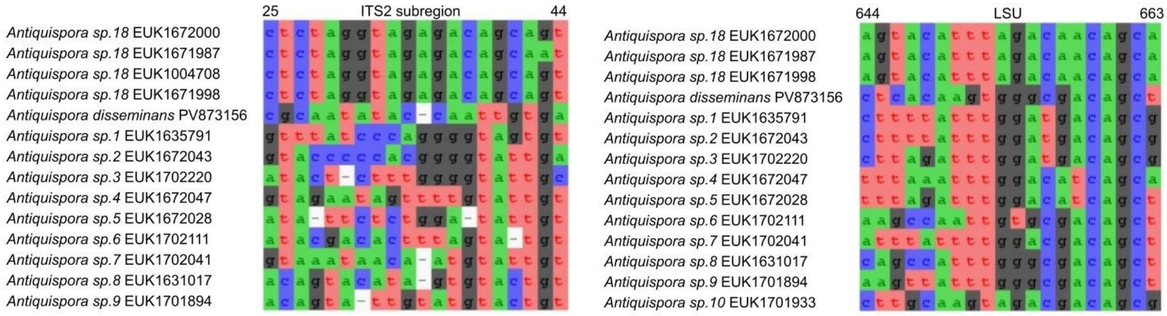

Figure S18. Separation of *Antiquispora sp.18* from other species of *Antiquispora* based on the ITS region (ITS2 positions 25–44 ctctaggtagagacagcagt; one mismatch allowed) and LSU (positions 644–663 agtacatttagacaacagca; no mismatch allowed).

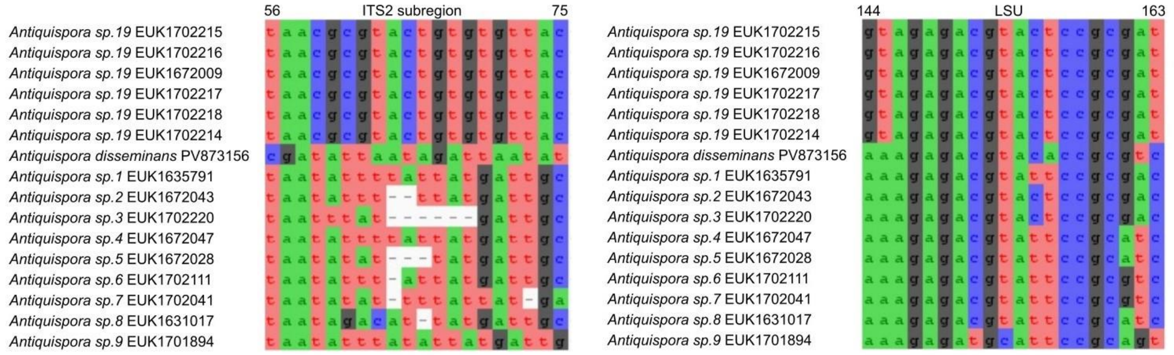

Figure S19. Separation of *Antiquispora sp.19* from other species of *Antiquispora* based on the ITS region (ITS2 positions 56–75 taacgcgtactgtgtgttac; no mismatch allowed) and LSU (positions 144–163 gtagagacgtactccgcgat; no mismatch allowed).

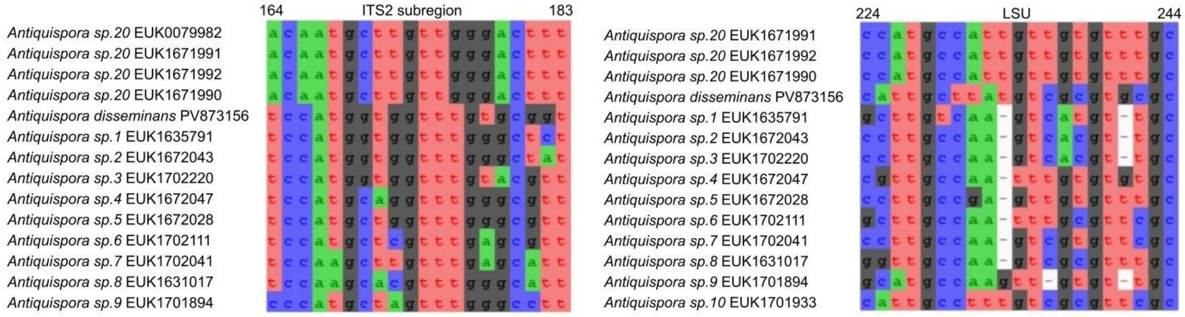

Figure S20. Separation of *Antiquispora sp.20* from other species of *Antiquispora* based on the ITS region (ITS2 positions 164–183 acaatgcttgttgggacttt; no mismatch allowed) and LSU (positions 224–244 ccatgccattgttgtgtttgc; no mismatch allowed).

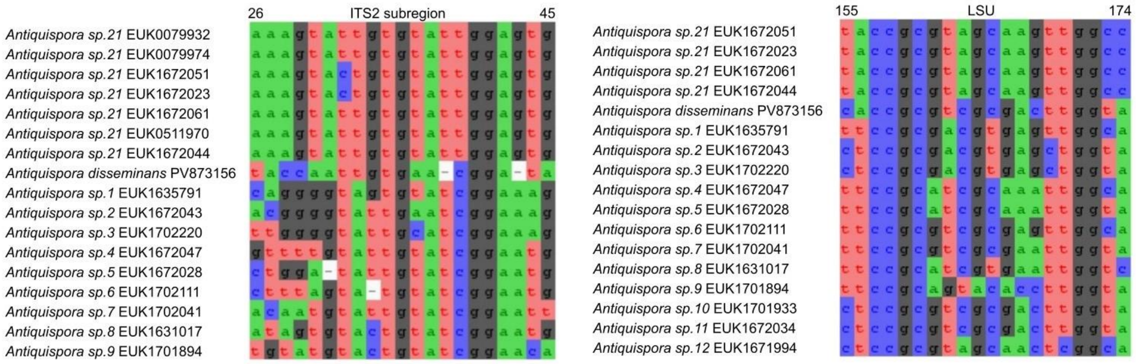

Figure S21. Separation of *Antiquispora sp.21* from other species of *Antiquispora* based on the ITS region (ITS2 positions 26–45 aaagtattgtgtattggagt; one mismatch allowed) and LSU (positions 155–174 taccgcgtagcaagttggcc; no mismatch allowed).

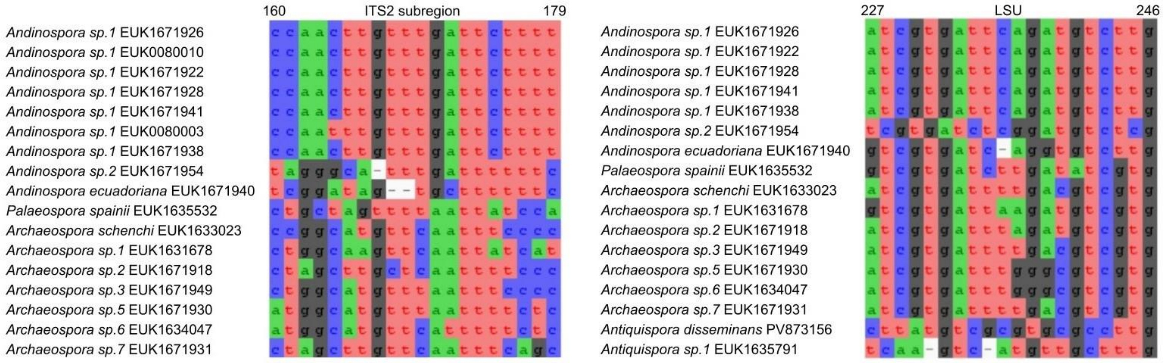

Figure S22. Separation of *Andinospora sp.1* from other species based on the ITS region (ITS2 positions 160–179 ccaacttgtttgattctttt; one mismatch allowed) and LSU (positions 227–246 atcgtgattcagatgtcttg; no mismatch allowed).

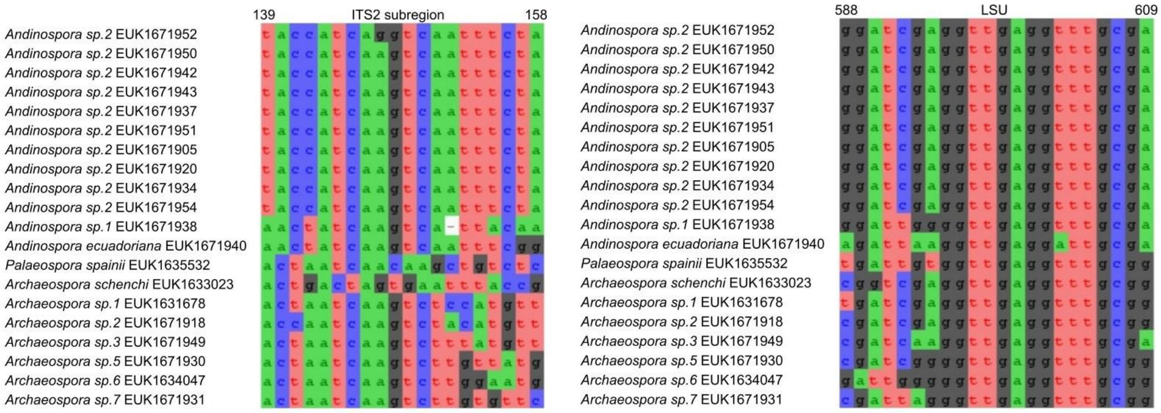

Figure S23. Separation of *Andinospora sp.2* from other species based on the ITS region (ITS2 positions 139–158 taccatcaagtcaatttcta; one mismatch allowed) and LSU (positions 588–609 ggatcgaggttgaggtttgcga; no mismatch allowed).

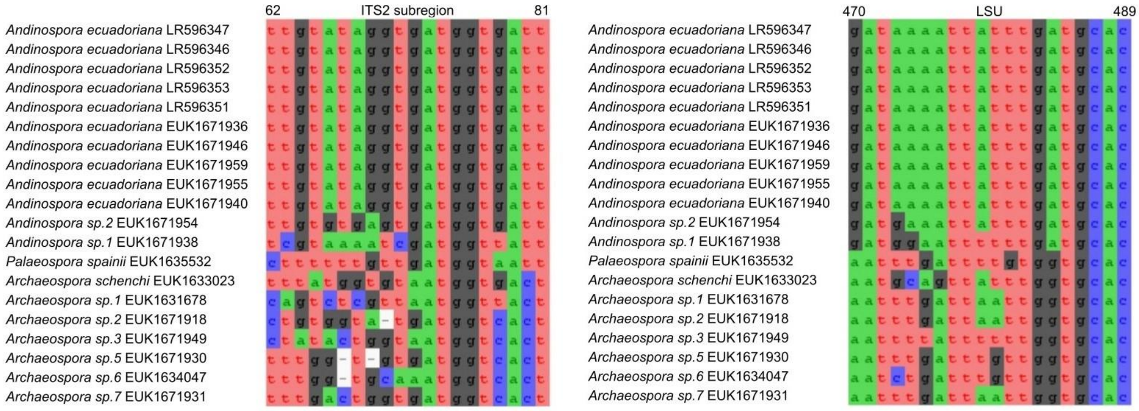

Figure S24. Separation of *Andinospora ecuadoriana* from other species based on the ITS region (ITS2 positions 62–81 ttgtataggtgatggtgatt; no mismatch allowed) and LSU (positions 470–489 gataaaattatttgatgcac; no mismatch allowed).

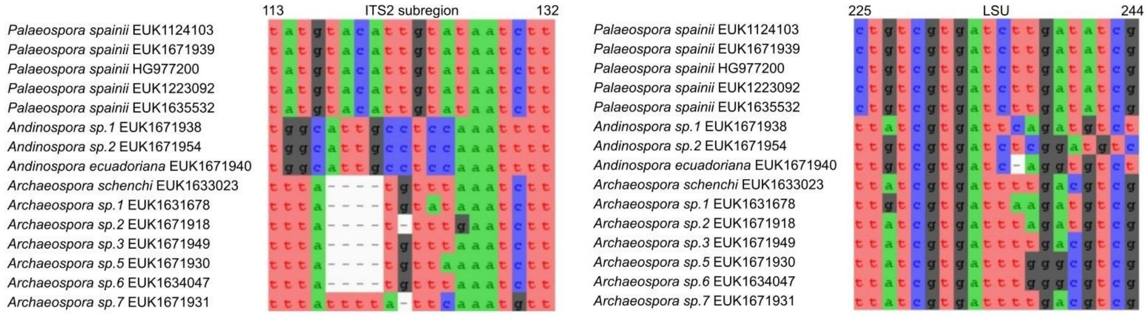

Figure S25. Separation of *Palaeospora spainii* from other species based on the ITS region (ITS2 positions 113–132 tatgtacattgtataatctt; no mismatch allowed) and LSU (positions 225–244 ctgtcgtgatcttgatatcg; no mismatch allowed).

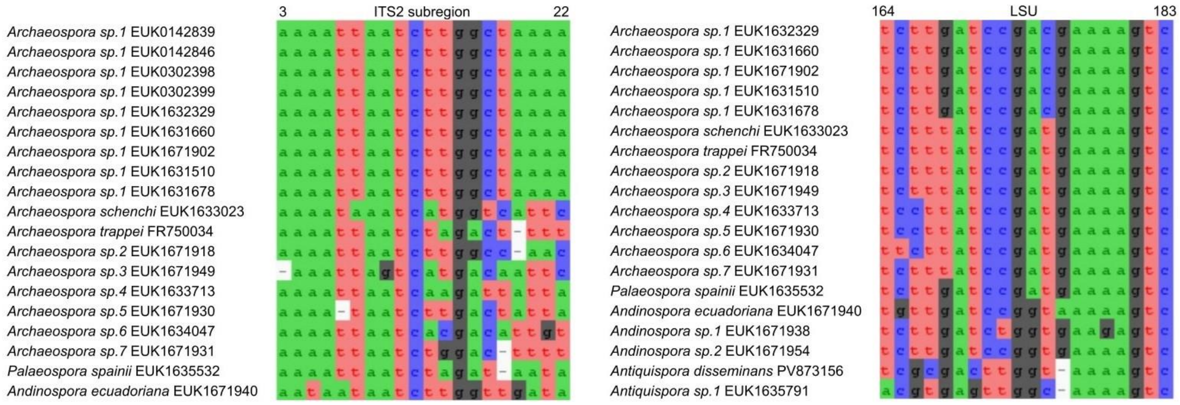

Figure S26. Separation of *Archaeospora sp.1* from other species based on the ITS region (ITS2 positions 3–22 aaaattaatcttggctaaaa; no mismatch allowed) and LSU (positions 164–183 tcttgatccgacgaaaagtc; no mismatch allowed).

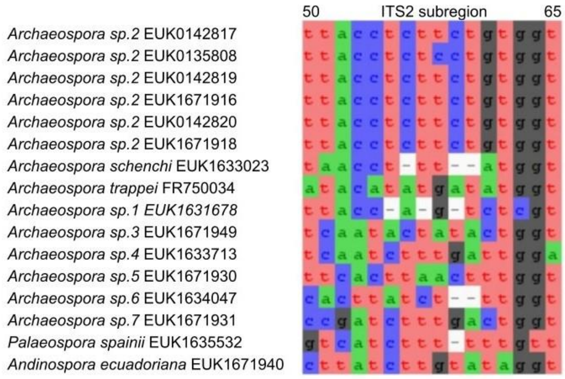

Figure S27. Separation of *Archaeospora sp.2* from other species based on the ITS region (ITS2 positions 50–65 ttacctcttctgtggt; one mismatch allowed).

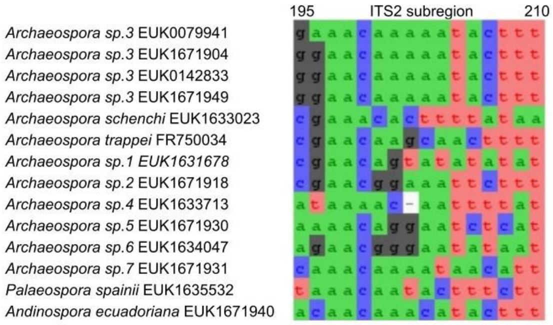

Figure S28. Separation of *Archaeospora sp.3* from other species based on the ITS region (ITS2 positions 195–210 ggaacaaaaatacttt; one mismatch allowed).

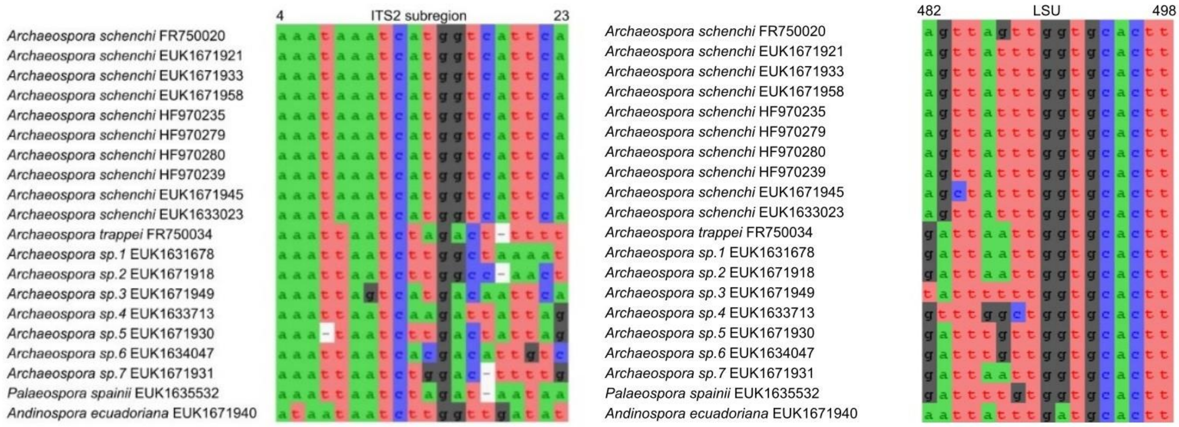

Figure S29. Separation of *Archaeospora schenchi* from other species based on the ITS region (ITS2 positions 4–23 aaataaatcatggtcattca; one mismatch allowed) and LSU (positions 482–498 agttatttggtgcactt; one mismatch allowed).

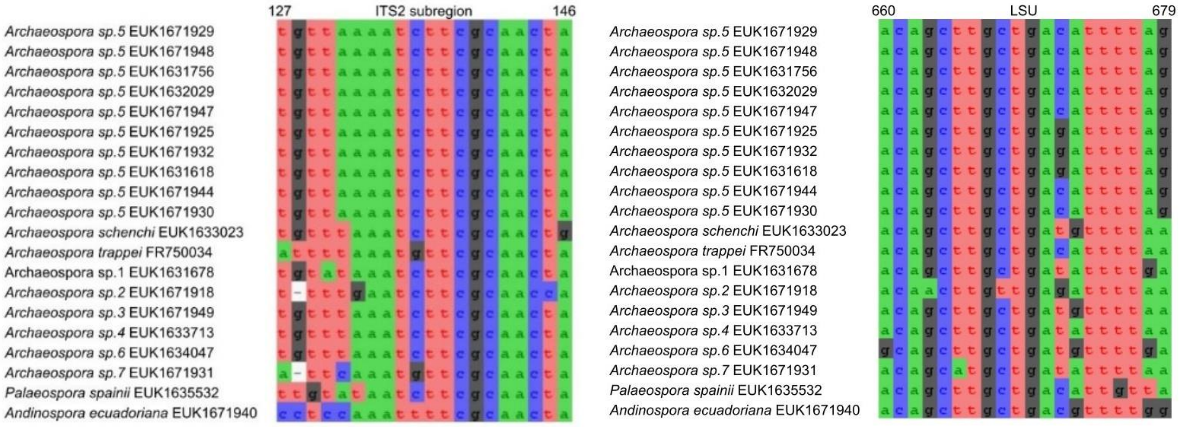

Figure S30. Separation of *Archaeospora sp.5* from other species based on the ITS region (ITS2 positions 127–146 tgttaaaatcttcgcaacta; no mismatch allowed) and LSU (positions 660–679 acagcttgctgacattttag; one mismatch allowed).

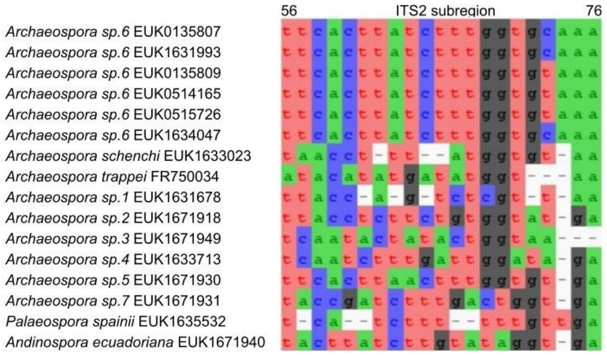

Figure S31. Separation of *Archaeospora sp.6* from other species based on the ITS region (ITS2 positions 56–76 ttcacttatctttggtgcaaa; one mismatch allowed).

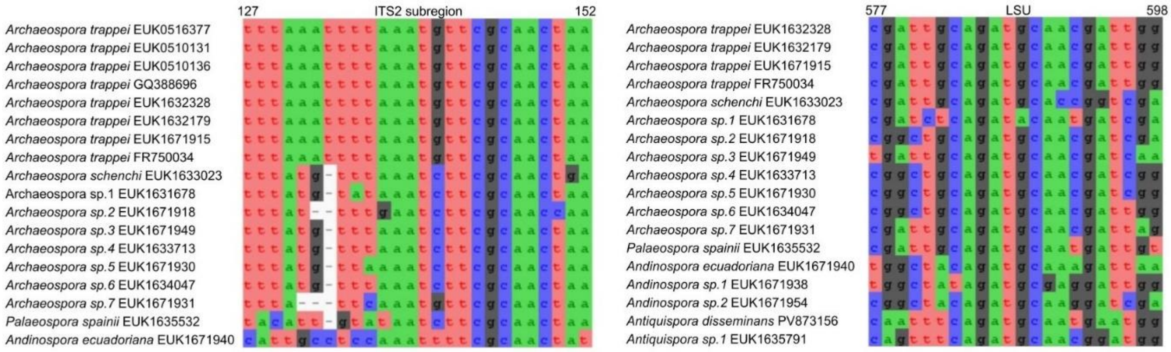

Figure S32. Separation of *Archaeospora trappei* from other species based on the ITS region (ITS2 positions 127–152 tttaaattttaaatgttcgcaactaa; one mismatch allowed) and LSU (positions 577–598 cgattgcagatgcaacgattgg; no mismatch allowed).

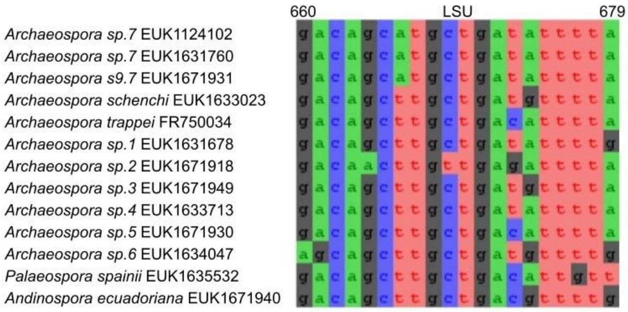

Figure S33. Separation of *Archaeospora sp.7* from other species based on the LSU (positions 660–679 gacagcatgctgatatttta; no mismatch allowed).
