# Supplementary material for: ﻿Morphological and phylogenetic analysis of the early-diverging lineage of Glomeromycota suggest two new genera and recombinations in Archaeosporales
Source: MycoKeys. 2025 Nov 3;124:249–73. doi: 10.3897/mycokeys.124.166449 (PMC12603645; doi:10.3897/mycokeys.124.166449)
Supplement: Supplementary material 5 — Occurrence of fungal genera in the family Archaeosporaceae across different biomes, based on EUKARYOME database records [file mycokeys-124-249-s005.pdf]

Occurrence of fungal genera in the family *Archaeosporaceae* across different biomes, based on EUKARYOME database records. The table summarizes the presence of each genus in natural and anthropogenic biomes, including forests, grasslands, shrublands, woodlands, deserts, and aquatic environments.

**Biomes**	*Antiquispora*	*Archaeospora*	*Andinospora*
**Anthropogenic**			
Cropland	x	x	
Rangeland	x	x	
Urban area	x	x	
Village area	x	x	
**Aquatic**			
Freshwater lake	x		
Freshwater river		x	
**Desert**			
Subtropical desert	x		
Montane desert		x	
Temperate desert		x	
**Forest**			
Mediterranean forest	x	x	
Subpolar coniferous forest	x	x	
Subtropical broadleaf forest	x	x	x
Subtropical coniferous forest	x	x	
Tropical broadleaf forest	x	x	x
Tropical coniferous forest	x	x	x
**Grassland**			
Flooded grassland	x	x	
Montane grassland	x	x	
Temperate grassland	x	x	x
**Shrubland**			
Montane shrubland	x		
Subtropical shrubland	x		
Temperate shrubland	x	x	
Tropical shrubland	x		
Xeric shrubland	x	x	
**Tundra**	x		
**Woodland**			
Subtropical woodland	x		
Temperate woodland	x	x	x
Tropical woodland	x	x	x
